# Synthesis and Antioxidant Properties of HeteroBisNitrones Derived from Benzene Dicarbaldehydes

**DOI:** 10.3390/antiox11081575

**Published:** 2022-08-15

**Authors:** Daniel Diez-Iriepa, Isabel Iriepa, Francisco López-Muñoz, José Marco-Contelles, Dimitra Hadjipavlou-Litina

**Affiliations:** 1 Laboratory of Medicinal Chemistry, Institute of Organic Chemistry (CSIC), Juan de la Cierva 3, 28006 Madrid, Spain; 2 Departamento de Química Orgánica y Química Inorgánica, Universidad de Alcalá, Ctra. Madrid-Barcelona km 33.6, Alcalá de Henares, 28871 Madrid, Spain; 3 Institute of Chemical Research Andrés M. del Río, Alcalá University, Alcalá de Henares, 28871 Madrid, Spain; 4 Faculty of Health, Camilo José Cela University of Madrid (UCJC), 28692 Madrid, Spain; 5 Neuropsychopharmacology Unit, “Hospital 12 de Octubre” Research Institute, 28041 Madrid, Spain; 6 Centre for Biomedical Network Research on Rare Diseases (CIBERER), CIBER, ISCIII, 46010 Madrid, Spain; 7 Department of Pharmaceutical Chemistry, School of Pharmacy, Faculty of Health Sciences, Aristotle University of Thessaloniki, 54124 Thessaloniki, Greece

**Keywords:** antioxidants, free radical scavengers, HeteroBisnitrones, nitrones, PBN, preparation

## Abstract

We report herein the synthesis and antioxidant profile of nine novel heterobisnitrones (hBNs) as new *α*-phenyl-*tert*-butylnitrone (PBN) analogues. The synthesized hBNs 1–9 were evaluated for their antioxidant activity using different in vitro techniques, while they were also tested as inhibitors of soybean LOX, as an indication of their anti-inflammatory effect. Nitrone hBN9 is the most potent antioxidant presenting higher anti-lipid peroxidation and hydroxyl radicals scavenging activities as well as higher lipoxygenase inhibition. In silico calculations reveal that hBN9 follows Lipinski’s rule of five and that the molecule is able to penetrate theoretically the brain. All these results led us to propose hBN9 as a new potent antioxidant nitrone.

## 1. Introduction

Among neuroscientists around the world, there is an overwhelming consensus pointing to oxidative stress as one of the key biological events at the origin of a number of human conditions, including stroke, Alzheimer’s (AD) and Parkinson’s disease (PD) [1].

Nowadays stroke patients are being exclusively treated in the clinics by recombinant tissue plasminogen activator (rtPA) [2], a drug showing serious secondary effects [3] and restricted administration periods of time for an effective cure [4]. Thus, a more tolerated and efficient therapy for stroke is being investigated in different laboratories, putting the focus on the control and trapping of highly toxic oxygenated radical species [5]. AD is a progressive irreversible disorder [6], typified by massive and significant neuronal death [7]. PD is a chronic, neurodegenerative condition affecting 1% of the elderly over age 60 [8], and caused by the deficit of dopamine, the origin of the well-known symptoms associated with this pathology: tremor, rigidity, bradykinesia, and postural instability [9]. To date, all approved therapies for the therapy of PD target to enhance striatal dopamine levels [10].

**Figure 1:**
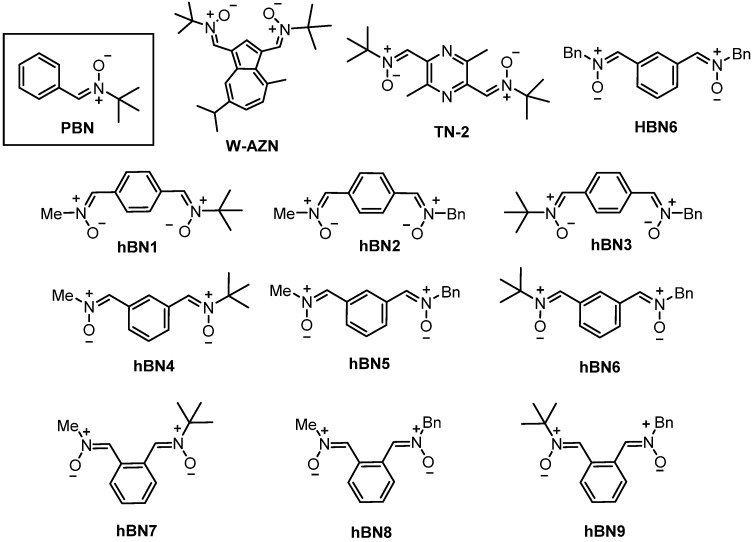
Structures of PBN, W-AZN, TN-2, HBN6, HTN3 and hBNs 1–9.

In this context, synthetic and antioxidant nitrones have been historically selected, analyzed and applied to cure stroke, and neurodegenerative diseases [11]. This is the case of *α*-phenyl *N*-*tert*-butylnitrone (PBN) (Figure 1), a simple and reputed drug for preventing and reversing cerebral ischemia in suitable animal models [12]. The permanent interest in nitrones for the potential therapy of stroke is very well documented in the current literature [13], our research team is strongly concerned and involved in diverse projects targeted to find new and more efficient nitrones for the improved treatment of stroke [14].

In this context, we have been attracted by the properties and therapeutic possibilities of bis-nitrones such as azulenyl nitrone W-AZN (Figure 1) [15,16] and bis-nitrone TN-2 (Figure 1), [17] and as consequence, we have recently reported a series of homobisnitrones (HBNs) related to PBN, from which HBN6 (Figure 1) exhibited good antioxidant properties [18].

Based on the neuroprotective results observed for HBN6 [18] (Figure 1), we were also interested to check if diversely substituted *N*,*N*′-dialkylbisnitrones, such as heterobisnitrones (hBNs), would afford antioxidant and biological activities compared to the previously described HBNs [18]. Consequently, we designed and synthesized hBNs 1–9 (Figure 1), by combining the *N*-Bn motif with *N*-Me and *N*-*tert*-Bu, respectively, in similar *para, meta* and *ortho* arrangements. Here we report their synthesis and evaluation as antioxidant agents.

## 2. Materials and Methods

### 2.1. General Methods

Compound purification was performed by column chromatography with Merck Silica Gel (40–63 µm) or by flash chromatography (Biotage Isolera One equipment) and the adequate eluent for each case. The reaction course was monitored by thin layer chromatography (t.l.c.), revealing with UV light (λ = 254 nm) and ethanolic solution of vanillin or ninhydrin. Melting points were determined using a Reichert Thermo Galen Kofler block and are uncorrected. Samples were dissolved in CDCl_3_ or DMSO-*d_6_* using TMS as an internal standard for ^1^H NMR spectra. In ^13^C NMR spectra, CDCl_3_ central signal (77.0 ppm) and DMSO-*d* (39.5 ppm) were used as references. ^1^H NMR and ^13^C NMR spectra were obtained in Bruker Avance 300 (300 MHz) and Bruker Avance 400 III HD (400 Hz) spectrometers. Chemical shifts (δ) are given in ppm. Coupling constants (*J*) are given in Hz. Signal multiplicity is abbreviated as singlet (s), doublet (d), or multiplet (m). IR spectra were recorded on a Perkin-Elmer Spectrum One B spectrometer. Units are cm^−1^. Low-resolution mass spectra were recorded on an Agilent HP 1100 LC/MS Spectrometer, whereas High-Resolution mass spectrometry (Exact Mass) was performed in an AGILENT 6520 Accurate-Mass QTOF LC/MS Spectrometer. Elemental analysis was performed in an Elementary Chemical Analyzer LECO CHNS-932. For the biological assays the following materials were used: Nordihydroguaiaretic acid (NDGA), Trolox, 2,2′-azobis(2-amidinopropane) dihydrochloride (AAPH), Soybean LOX linoleic acid sodium salt was purchased from the Aldrich Chemical Co. Milwaukee, WI, (USA). Phosphate buffer (0.1 M and pH 7.4) was prepared by mixing an aqueous KH_2_PO_4_ solution (50 mL, 0.2 M), and an aqueous NaOH solution (78 mL, 0.1 M); the pH (7.4) was adjusted by adding a solution of KH_2_PO_4_ or NaOH). For the in vitro tests a Lambda 20 (Perkin–Elmer-PharmaSpec 1700) UV–Vis double beam spectrophotometer was used.

#### 2.1.1. General Method for the Synthesis of hBNs 1–9

To a suspension of the appropriate benzene dicarbaldehyde (1 mmol) in dry THF (10 mL), anhydrous NaHCO_3_ (2 equiv), Na_2_SO_4_ (2 equiv) and the corresponding N-alkylhydroxylamine hydrochloride (1.2 equiv) were added. The mixture was stirred at room temperature (rt), for the time indicated in each case. Then, the other N-alkylhydroxylamine hydrochloride (1.2 equiv) and NaHCO_3_ (2 equiv) were added to the mixture and were stirred at rt or irradiated at 90 °C, and 15 bar. Finally, the solvent was removed, and the crude was purified by column chromatography.

##### (Z)-N-tert-Butyl-1-(4-((Z)-(methyloxidoazaneylidene)methyl)phenyl)methanimine oxide (hBN1)

Following the general Method, a suspension of terephthalaldehyde (134 mg, 1 mmol), anhydrous NaHCO_3_ (168 mg, 2 mmol), Na_2_SO_4_ (284 mg, 2 mmol) and *N*-*tert*-butylhydroxylamine hydrochloride (150.7 mg, 1.2 mmol), in dry THF (10 mL), was stirred at rt for 1 d. Then, *N*-methylhydroxylamine hydrochloride (100.2 mg, 1.2 mmol) and anhydrous NaHCO_3_ (84 mg, 1 mmol) were added and the mixture was stirred for 2 d. After work-up and purification by column chromatography eluting with MeOH:CH_2_Cl_2_ 3%, hBN1 (47 mg, 20%) was isolated: mp 115–117 °C; IR (KBr) ν 3460, 2978, 1577, 1413, 1169 cm^−1^; ^1^H NMR (400 MHz, DMSO-d_6_) δ 8.37 (d, *J* = 8.6 Hz, 2 H), 8.23 (d, *J* = 8.6 Hz, 2 H), 7.88 (s, 1 H), 7.86 (s, 1 H), 3.79 (s, 3 H), 1.51 (s, 9 H); ^13^C NMR (101 MHz, DMSO-d_6_) δ 134.0, 132.9, 132.1, 128.9, 128.5 (2 C), 127.8 (2 C), 71.1, 54.6, 28.3 (3C); EM (ES) *m/z* (%): 269 [M+1]^+^, 291 [M+Na]^+^. HRMS (ESI_ACN) Calcd. for C_13_H_18_N_2_O_2_: 234.1368. Found: 234.1369 Anal. Calcd. for C_13_H_18_N_2_O_2_.1/4 H_2_O: C, 65.39; H, 7.81; N, 11.73. Found: C, 65.35; H, 7.82; N, 11.57.

##### (Z)-N-Benzyl-1-(4-((Z)-methyloxidoazaneylidene)methyl)phenyl)methanimine oxide (hBN2)

Following the general Method, a suspensión of terephthalaldehyde (134 mg, 1 mmol), anhydrous NaHCO_3_ (168 mg, 2 mmol), Na_2_SO_4_ (284 mg, 2 mmol) and *N*-benzylhydroxylamine hydrochloride (190.8 mg, 1.2 mmol), in dry THF (10 mL), was stirred at rt for 1 d. Then, *N*-methylhydroxylamine hydrochloride (100.2 mg, 1.2 mmol) and anhydrous NaHCO_3_ (84 mg, 1 mmol) were added and the mixture was stirred at rt for 2 d. After work-up and purification by column chromatography eluting with AcOEt, hBN2 (50 mg, 19%) was isolated: mp 191-193 °C; IR (KBr) ν 3420, 1579, 1423, 1168 cm^−1^; ^1^H NMR (400 MHz, DMSO-d_6_) δ 8.24 (s, 4 H), 8.12 (s, 1 H), 7.86 (s, 1 H), 7.61–7.47 (m, 2 H), 7.49–7.31 (m, 3 H), 5.09 (s, 2 H), 3.79 (s, 3 H); ^13^C NMR (101 MHz, DMSO-d_6_) δ 135.1, 134.0, 133.4, 132.5, 132.2, 129.4 (2 C), 128.9 (2 C), 128.8, 128.2 (2 C), 128.0 (2 C), 70.6, 54.7; MS (ES) *m/z* (%): 269 [M+1]^+^, 291 [M+Na]^+^. HRMS (ESI_ACN) Calcd. for C_16_H_16_N_2_O_2_: 268.1212. Found: 268.1205. Anal. Calcd. for C_16_H_16_N_2_O_2_: C, 71.62; H, 6.01; N, 10.44. Found: C, 71.28; H, 6.10; N. 10.35.

##### (Z)-N-Benzyl-1-(4-((Z)-(tert-butyloxidoazaneylidene)methyl)phenyl)methanimine oxide (hBN3)

Following the general Method, a suspension of terephthalaldehyde (134 mg, 1 mmol), anhydrous NaHCO_3_ (168 mg, 2 mmol), Na_2_SO_4_ (284 mg, 2 mmol) and *N*-benzylhydroxylamine hydrochloride (190.8 mg, 1.2 mmol), in dry THF absolute (10 mL), was stirred at rt for 1 d. Then, *N*-*tert*-butylhydroxylamine hydrochloride (150.7 mg, 1.2 mmol) and anhydrous NaHCO_3_ (84 mg, 1 mmol) were added, and the mixture was irradiated for 2 h. After work-up and purification by column chromatography eluting with MeOH:CH_2_Cl_2_ 3%, hBN3 (78 mg, 25%) was isolated: mp 170-172 °C; IR (KBr) ν 3435, 2977, 1573,1362, 1126 cm^−1^; ^1^ H NMR (400 MHz, DMSO-d_6_) δ 8.35 (d, *J* = 8.9 Hz, 2 H), 8.21 (d, *J* = 8.9 Hz, 2 H), 8.09 (s, 1 H), 7.86 (s, 1 H), 7.50–7.47 (m, 2 H), 7.40–7.34 (m, 3 H), 5.06 (s, 2 H), 1.48 (s, 9 H); ^13^C NMR (101 MHz, DMSO-d_6_) δ 135.1, 133.4, 133.0, 132.0, 129.5, 129.4, 128.9 (2 C), 128.8, 128.5 (2 C), 128.4 (2 C), 128.0, 71.2, 70.6, 28.3 (3 C); MS (ES) *m/z* (%): 311 [M+1]^+^, 333 [M+Na]^+^. HRMS (ESI_ACN) Calcd. for C_19_H_22_N_2_O_2_: 310.1681. Found: 310.1680. Anal. Calcd. for C_19_H_22_N_2_O_2_: C, 73.52; H, 7.14; N, 9.03. Found: C, 73.16; H, 7.13; N, 9.20.

##### (Z)-N-tert-Butyl-1-(3-((Z)-(methyloxidoazaneylidene)methyl)phenyl)methanimine oxide (hBN4)

A suspension of isophthalaldehyde (268 mg, 2 mmol), anhydrous NaHCO_3_ (336 mg, 4 mmol), Na_2_SO_4_ (568 mg, 4 mmol) and *N*-*tert*-butylhydroxylamine hydrochloride (301.4 mg, 2.4 mmol), in dry THF (20 mL), was stirred at rt for 3 d. Then, *N*-methylhydroxylamine hydrochloride (200.4 mg, 2.4 mmol) and anhydrous NaHCO_3_ (168 mg, 2 mmol) were added and the mixture was stirred for 1 d. After work-up and purification by column chromatography eluting with DCM/MeOH 3%, hetero-bis-nitrone hBN4 (216 mg, 46%) was isolated: mp 114-116 °C; IR (KBr) ν 3042, 2997, 1561, 1422, 1119 cm^−1^; ^1^H NMR (500 MHz, CDCl_3_) δ 9.06 (t, *J* = 1.4 Hz, 1H), 8.46 (dt, *J* = 7.9, 1.4 Hz, 1H), 8.32 (dt, *J* = 7.9, 1.4 Hz, 1H), 7.60 (s, 1H), 7.47 (t, *J* = 7.9 Hz, 1H), 7.41 (s, 1H), 3.87 (d, *J* = 0.7 Hz, 3H), 1.60 (s, 9H); ^13^C NMR (126 MHz, CDCl_3_) δ 135.0, 131.2, 130.5, 130.3, 129.7, 129.6, 128.9, 128.7, 71.1, 54.5, 28.3 (3 C). HRMS (ESI_ACN) Calcd. for C_13_H_18_N_2_O_2_: 234.1368. Found: 234.1373. Anal. Calcd. for C_13_H_18_N_2_O_2_.1/4 H_2_O: C, 65.39; H, 7.81; N, 11.73. Found: C, 65.78; H, 7.74; N, 11.60.

##### (Z)-N-Benzyl-1-(3-((Z)-methyloxidoazaneylidene)methyl)phenyl)methanimine oxide (hBN5)

A suspension of isophthalaldehyde (268 mg, 2 mmol), anhydrous NaHCO_3_ (336 mg, 4 mmol), Na_2_SO_4_ (568 mg, 4 mmol) and *N*-benzylhydroxylamine hydrochloride (381.6 mg, 2.4 mmol), in dry THF (20 mL), was stirred at rt for 1 d. Then, *N*-methylhydroxylamine hydrochloride (200.5 mg, 2.4 mmol) and anhydrous NaHCO_3_ (168 mg, 2 mmol) were added and the mixture was stirred at rt for 2 d. After work-up and purification by column chromatography eluting with DCM/MeOH 3%, hetero-bis-nitrone hBN5 (235.4 mg, 44%) was isolated as a mixture of isomers in a 1:1 ratio, that we were unable to separate and were submitted together to biological evaluation: mp 146-148 °C; IR (KBr) ν 3068, 1571, 1415, 1140 cm^−1^; ^1^H NMR (400 MHz, CDCl_3_) δ 8.89 (s, 1H), 8.32–8.25 (m, 2H), 7.47–7.29 (m, 8H), 4.99 and 4.97 (two s, 2H), 3.82 and 3.80 (two s, 3H). HRMS (ESI_ACN) Calcd. for C_16_H_16_N_2_O_2_: 268.1211. Found: 268.1219. Anal. Calcd. for C_16_H_16_N_2_O_2_ 1/6 H_2_O: C, 70.83; H, 6.07; N, 10.33. Found: C, 70.78; H, 6.04; N, 10.07.

##### (Z)-N-Benzyl-1-(3-((Z)-(tert-butyloxidoazaneylidene)methyl)phenyl)methanimine oxide (hBN6)

A suspension of isophthalaldehyde (268 mg, 2 mmol), anhydrous NaHCO_3_ (168 mg, 2 mmol), Na_2_SO_4_ (284 mg, 2 mmol) and *N*-benzylhydroxylamine hydrochloride (381.6 mg, 2.4 mmol), in dry THF (20 mL), was stirred at rt for 1 d. Then, *N*-*tert*-butylhydroxylamine hydrochloride (301.4 mg, 2.4 mmol) and anhydrous NaHCO_3_ (168 mg, 2 mmol) were added, and the mixture was irradiated for 2 h. After work-up and purification by column chromatography eluting with hexane:AcOEt 2/3, hetero-bis-nitrone hBN6 (213.5 mg, 34%) was isolated: mp 123-125 °C; IR (KBr) ν 3076, 2976, 1576,1362, 1170 cm^−1^; ^1^H NMR (400 MHz, CDCl_3_) δ 9.09 (s, 1H), 8.39–8.34 (m, 2H), 7.57 (s, 1H), 7.47–7.38 (m, 7H), 5.04 (s, 2H), 1.58 (s, 9H); ^13^C NMR (101 MHz, CDCl_3_) δ 134.3, 133.0, 131.2, 130.6 (2 C), 130.08 (2 C), 129.9 (2 C), 129.5, 129.3, 129.2, 129.2, 128.8, 71.5, 71.2, 28.4 (3 C). HRMS (ESI_ACN) Calcd. for C_19_H_22_N_2_O_2_: 310.1681. Found: 310.1689. Anal. Calcd. for C_19_H_22_N_2_O_2_: C, 73.52; H, 7.14; N, 9.03. Found: C, 73.81; H, 7.65; N, 8.62.

##### (Z)-N-tert-Butyl-1-(2-((Z)-(methyloxidoazaneylidene)methyl)phenyl)methanimine oxide (hBN7)

A suspension of phthalaldehyde (268 mg, 2 mmol), anhydrous NaHCO_3_ (336 mg, 4 mmol), Na_2_SO_4_ (568 mg, 4 mmol) and *N*-*tert*-butylhydroxylamine hydrochloride (301.4 mg, 2.4 mmol), in dry THF (20 mL), was stirred at rt for 3 d. Then, *N*-methylhydroxylamine hydrochloride (200.4 mg, 2.4 mmol) and anhydrous NaHCO_3_ (168 mg, 2 mmol) were added and the mixture was stirred for 1 d. After work-up and purification by column chromatography eluting with DCM/MeOH 2%, hetero-bis-nitrone hBN7 (131.4 mg, 56%) was isolated: mp 130-132 °C; IR (KBr) ν 3035, 2975, 1570, 1393 cm^−1^; ^1^H NMR (500 MHz, CDCl_3_) δ 8.54–8.48 (m, 1H), 8.46–8.41 (m, 1H), 7.71 (s, 1H), 7.49 (s, 1H), 7.47–7.41 (m, 2H), 3.90 (d, *J* = 0.7 Hz, 3H), 1.63 (s, 9H); ^13^C NMR (126 MHz, CDCl_3_) δ 133.1, 129.9, 129.5, 128.9, 128.6, 128.5, 128.4, 127.5, 71.3, 54.7, 28.4 (3C). HRMS (ESI_ACN) Calcd. for C_13_H_18_N_2_O_2_: 234.1368. Found: 234.1368. Anal. Calcd. for C_13_H_18_N_2_O_2_: C, 66.64; H, 7.74; N, 11.96. Found: C, 66.62; H, 7.78; N, 11.79.

##### (Z)-N-Benzyl-1-(2-((Z)-(methyloxidoazaneylidene)methyl)phenyl)methanimine oxide (hBN8)

A suspensión of phthalaldehyde (268 mg, 2 mmol), anhydrous NaHCO_3_ (336 mg, 4 mmol), Na_2_SO_4_ (568 mg, 4 mmol) and *N*-benzylhydroxylamine hydrochloride (381.6 mg, 2.4 mmol), in dry THF (20 mL), was stirred at rt for 1 d. Then, *N*-methylhydroxylamine hydrochloride (200.5 mg, 2.4 mmol) and anhydrous NaHCO_3_ (168 mg, 2 mmol) were added and the mixture was stirred at rt for 2 d. After work-up and purification by column chromatography eluting with DCM/MeOH 3%, hetero-bis-nitrone hBN8 (266.6 mg, 50%) was isolated: mp 142-144 °C; IR (KBr) ν 3368, 2972, 1578, 1437, 1175 cm^−1^; ^1^H NMR (500 MHz, CDCl_3_) δ 8.61–8.56 (m, 2H), 7.53–7.51 (m, 3H), 7.45–7.43 (m, 5H), 7.28 (s, 1H), 5.09 (s, 2H), 3.76 (s, 3H); ^13^C NMR (126 MHz, CDCl_3_) δ 133.1, 132.1, 131.4, 130.0, 129.9, 129.4, 129.2 (2 C), 129.0, 128.4 (2 C), 128.3, 128.3, 128.1, 71.4, 54.6. HRMS (ESI_ACN) Calcd. for C_16_H_16_N_2_O_2_: 268.1211. Found: 268.1217. Anal. Calcd for C_16_H_16_N_2_O_2_ 7/8: C, 67.65; H, 6.30; N, 9.86. Found: C, 67.75; H, 6.15; N, 9.62.

##### (Z)-N-Benzyl-1-(2-((Z)-(tert-butyloxidoazaneylidene)methyl)phenyl)methanimine oxide (hBN9)

A suspension of phthalaldehyde (268 mg, 2 mmol), anhydrous NaHCO_3_ (168 mg, 2 mmol), Na_2_SO_4_ (284 mg, 2 mmol) and *N*-benzylhydroxylamine hydrochloride (381.6 mg, 2.4 mmol), in dry THF (20 mL), was stirred at rt for 1 d. Then, *N*-*tert*-butylhydroxylamine hydrochloride (301.4 mg, 2.4 mmol) and anhydrous NaHCO_3_ (168 mg, 2 mmol) were added, and the mixture was irradiated for 2 h. After work-up and purification by column chromatography eluting with hexane:AcOEt 1/1, hetero-bis-nitrone hBN9 (154 mg, 25%) was isolated: mp 114-116 °C; IR (KBr) ν 3435, 2977, 1576, 1357, 1153 cm^−1^; ^1^H NMR (500 MHz, CDCl_3_) δ 8.69–8.65 (m, 1H), 8.58–8.54 (m, 1H), 7.52–7.49 (m, 2H), 7.47–7.40 (m, 6H), 7.39 (s, 1H), 5.08 (s, 2H), 1.45 (s, 9H); ^13^C NMR (126 MHz, CDCl_3_) δ 132.9, 131.4, 129.9 (2 C), 129.8 (2 C), 129.5, 129.3, 129.2, 129.0, 128.4, 128.3, 128.3, 126.6, 71.3, 71.5, 28.1 (3 C). HRMS (ESI_ACN) Calcd. for C_19_H_22_N_2_O_2_: 268.1211. Found: 268.1219. Anal. Calcd for C_19_H_22_N_2_O_2_: C, 73.52; H, 7.14; N, 9.03. Found: C, 73.16; H, 7.13; N, 9.20.

### 2.2. Estimation of Lipophilicity as Clog P

Lipophilicity is an important physicochemical property related to biological activity and ADME properties. Thus, we used Bioloom of Biobyte Corp for the theoretical calculation of lipophilicity as Clog *p* values (BioByte Home Page. Available online: http://www.biobyte.com) (accessed on 1 July 2022).

### 2.3. Biological Antioxidant Assays Used for the Study of hBNs 1–9 and PBN

We used different types of assays to measure in vitro antioxidant activity of nitrones, such as the inhibition of lipid peroxidation (LP) induced by AAPH, the DMSO method for the hydroxyl radical scavenging activity, the ABTS^+∙^–decolourization assay and the in vitro inhibition of soybean lipoxygenase (LOX).

#### 2.3.1. Inhibition of Linoleic Acid Peroxidation

10 μL of a 16 mM linoleate sodium solution and 0.93 mL of 0.05 M phosphate buffer (pH 7.4), prethermostated at 37 °C, were incorporated in the UV cuvette. Then, 50 μL of 40 mM AAPH solution was added and used as a free radical initiator at 37 °C under air [18]. Finally, 10 μL of the tested compounds were added. The oxidation of linoleic acid sodium salt results in a conjugated diene hydroperoxide. The reaction was monitored at 234 nm and the absorbance values were recorded. Trolox was used as a reference compound and positive control.

#### 2.3.2. In Vitro Inhibition of Soybean Lipoxygenase (LOX)

The in vitro study was performed as reported previously by our group. [18] The tested compounds were incubated at room temperature with sodium linoleate (0.1 mM) and 0.2 mL of enzyme solution (1/9 × 10^−4^ *w*/*v* in saline). The method was based on the conversion of sodium linoleate to 13-hydroperoxylinoleic acid at 234 nm. NDGA (IC_50_ = 0.45 µM) was used as a standard (positive control). In order to determine the IC_50_ values, different concentrations were used. A blank determination was used first to serve as a negative control.

#### 2.3.3. Competition of the Tested Compounds with DMSO for Hydroxyl Radicals

The hydroxyl radicals were produced by the Fe^3+/^ascorbic acid system and detected by the determination of formaldehyde produced from the oxidation of DMSO. EDTA (0.1 mM), Fe^3+^ (167 μM), DMSO (33 mM) in phosphate buffer (50 mM, pH 7.4), the tested compounds (0.1 mM) and ascorbic acid (10 mM) were mixed in test tubes. [18] The solutions were incubated at 37 °C for 30 min. The reaction was stopped by CCl_3_COOH (17% *w/v*) and the % scavenging activity of the tested compounds for hydroxyl radicals was given. Trolox was used as a positive control.

#### 2.3.4. ABTS^∙+^–Decolorization Assay in Ethanolic Solution for Antioxidant Activity

In order to produce the ABTS radical cation (ABTS^+·^), ABTS stock solution in water (7 mM) was mixed with potassium persulfate (2.45 mM) and left in the dark at room temperature for 12–16 h before use. Our published experimental technique was used [18]. The results are recorded after 1 min of the mixing solutions at 734 nm. Trolox was used as a positive standard.

## 3. Results and Discussion

### 3.1. Chemistry

**Scheme 1:**
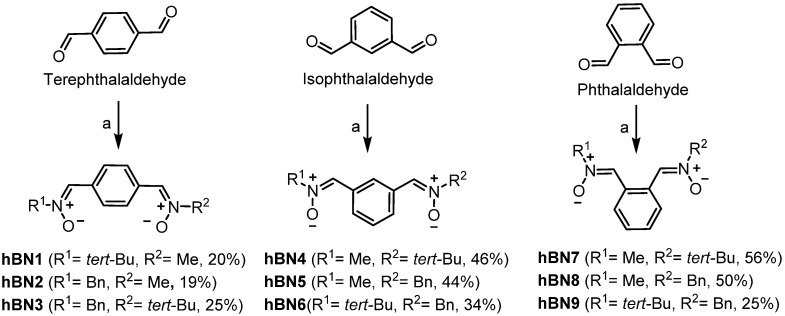
Synthesis of hBNs 1–9. Reagents and conditions: (a) 1. R^1^NHOH.HCl, NaHCO_3_, Na_2_SO_4_, rt, 24 h, THF. 2. R^2^NHOH.HCl, NaHCO_3_, Na_2_SO_4_, THF, rt [24 h (hBNs 4, 7) or 48 h (hBNs 1, 2, 5, 8)] or irradiated at 90 °C, and 15 bar, 2 h (hBNs 3, 6 and 9).

hBNs 1–9 have been prepared to start from commercially available terephthalaldehyde, isophthalaldehyde, and phthaladehyde in a “two-steps-one-pot-reaction”, under the same mild reaction conditions, by subsequent addition of the appropriate and selected *N*-alkylhydroxylamines, as shown in Scheme 1. The target bis-nitrones have been obtained as a pure compound showing correct IR, NMR and high-resolution mass spectra, as expected from their structures [18] (see Experimental Part and Supplementary Material).

### 3.2. Antioxidant Assays

The antioxidant power of hBNs 1–9, and PBN/HBN6, as reference molecules, on diverse antioxidant tests, using NDGA and Trolox as standards for comparative purposes, has been investigated in order to evaluate their capacity to block very reactive oxygen species (O_2_^−•^, HO^•^, HO_2_^•^), produced during the biochemical function of aerobic organisms able to injure biological targets such as lipids, proteins or DNA. Several different approaches are used to estimate the antioxidant activity of a compound, in order to obtain reproducible results.

The free radical initiator AAPH produces free radicals in the solution inducing linoleic acid’s oxidation, as a test to determine the antioxidant protection against lipid peroxidation in vitro. As shown in Table 1, hBN9 is the most potent antioxidant presenting the higher anti-lipid peroxidation activity. R^1^ = *tert*-Bu and R^2^ = Bn substituents are in the *ortho* position and this placement is related to the higher activity since hBN6 in which the same substituents are in the *meta* position does not present any activity. hBN3 the *para* derivative follows with an inhibition value of 60% lipid peroxidation. The combination of R^1^ = Me, and R^2^ = Bn gives significant results only for the *para* analogue hBN2, while no activity was given by the *ortho* and *meta* analogues. For the combination R^1^ = Me, R^2^ = *tert*-Bu, hBN1 the *para* and hBN7 the *ortho* analogue offer similar anti-lipid peroxidation activities (43 % and 41.5%). A perusal of the anti-lipid peroxidation results leads to the observation that comparing the PBN and HBN6, the best substituents combination is the one of hBN9 in the *ortho* position.

Next, we analyzed the ability of hBNs 1–9 to scavenge hydroxyl free radicals (HO^•^), possibly the most harmful among the reactive oxygen species (ROS), by determining their competition with DMSO for HO^•^. As shown in Table 1, considering the substituents combination and position we realized that for R^1^ = Me, R^2^ = Bn, the values range hBN7 > hBN4 > hBN1 (100 > 91 >43). For the series R^1^ = Me, R^2^ = Bn, the best activity was shown by hBN5 followed by hBN8 and hBN2, whereas for R^1^ = *tert*-Bu and R^2^ = Bn, hBN9 > hBN6, hBN2. Low lipophilicity values seem to be important for the hydroxyl radical scavenging activity.

Very interestingly, and as shown in Table 1, hBNs 1–9 did not show any interest in the ABTS radical cation (ABTS^•+^) decolourization assay.

Finally, and as shown in Table 1, among all the hBNs 1–9, the two most potent lipoxygenase (LOX) inhibitors were the two *ortho* derivatives hBN9 > hBN8. No role for lipophilicity was found. The inhibition presented by these two compounds will therapeutically offer in stroke or neurodegeneration.

## 4. Conclusions

In conclusion, in this work, we have reported the synthesis and antioxidant profile of hBNs 1–9. The bioassays’ results revealed that compound hBN9 is the most promising antioxidant agent (100% for antilipid peroxidation activity and 99.8% scavenging of hydroxyl radicals), as well as the most potent LOX inhibitor (IC_50_ = 57.5 μM). hBN8 is the second most potent analogue. Almost all nitrones are highly competitive DMSOs for hydroxyl radicals. Low lipophilicity seems to influence the hydroxyl scavenging activity. In silico calculations (Supporting Information) reveal that bis-nitrone hBN9 follows Lipinski’s rule of five and is able to penetrate theoretically the brain. These results pave the way for the bio-evaluation of target hBN9, the most potent antioxidant nitrone found here, in suitable in vitro/in vivo neuroprotection models for the therapy of stroke, and eventually other neurodegenerative diseases where oxidative stress has been identified to play a critical role.

## Figures and Tables

**Table 1:**
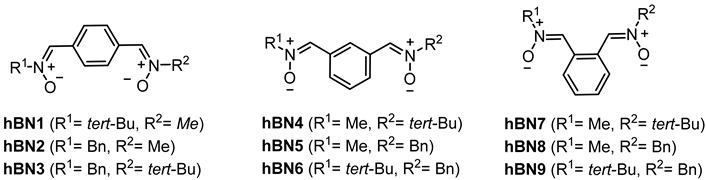
Antioxidant activity of hBNs 1–9 ^a^.

Nitrones/ Standard	ClogP ^b^	ILPO (%)	LOX Inhibition (% or IC_50_ [μM])	Scav. Activity for ^•^OH (%)	ABTS^+.^ (%)
** PBN **	3.02	11	23	no	5
**HBN6** [18]	** 4.96 **	** 37 **	** 29 **	** 81 **	No
** hBN1 **	0.04	43	11	43	no
** hBN2 **	0.69	48	nο	79	no
** hBN3 **	1.93	60	11	69	5
** hBN4 **	0.04	no	no	91	11
** hBN5 **	0.57	no	no	96.8	no
** hBN6 **	1.80	no	16	68.3	no
** hBN7 **	0.04	41.5	10	100	11.6
** hBN8 **	0.57	no	82.5 μM	89	7.6
** hBN9 **	** 1.80 **	** 100 **	** 57.5 ** **μM**	** 99.8 **	** 11.2 **
** NDGA **	3.92		0.45 μM		
** Trolox **	3.09	93		73	91

^a^ Nitrones tested at 100 µM. Values are means of three or four different determinations. no = no activity under the experimental conditions. Means within each column differ significantly (*p* < 0.05). ^b^ Biobyte BioByte Corporation, C-QSAR database, 201 W Fourth Str., Suite # 204, Claremont CA 91711-4707, USA.

## Data Availability

Not applicable.

## Supplementary Materials

The following supporting information can be downloaded at: https://www.mdpi.com/article/10.3390/antiox11081575/s1, Supporting Information: In silico determination of drug-likeness and IR, NMR and HRMS spectra of hBNs 1–9.

## Abbreviations

AAPH: 2:2′-azobis(2-amidinopropane) dihydrochloride; LOX, lipoxygenase; LP, lipid peroxidation; MTT, 3-(4,5-dimethylthiazol-2-yl)-2,5-diphenyltetrazolium bromide; NDGA, Nordihydroguaiaretic acid.
