# Supplementary material for: Synthesis and Antioxidant Properties of HeteroBisNitrones Derived from Benzene Dicarbaldehydes
Source: Antioxidants (Basel). 2022 Aug 15;11(8):1575. doi: 10.3390/antiox11081575 (PMC9404792; doi:10.3390/antiox11081575)
Supplement: Supplementary file 1 [file antioxidants-11-01575-s001.zip › antioxidants-1846468-supplementary.pdf]

Supplementary Material

Synthesis and Antioxidant Properties of HeteroBisNitrones Derived from Benzene Dicarbaldehydes

Daniel Diez-Iriepa, Isabel Iriepa, Francisco López-Muñoz, José Marco-Contelles,*

and Dimitra Hadjipavlou-Litina*

CONTENTS

1. In Silico Determination of Drug-Likeness of hBNs 1-9…………S2
2. NMR spectra of hBNs 1-9...…………………………………………S3-S32

1. In Silico Determination of Drug-Likeness of hBNs 1-9

Drug discovery is highly guided in the early steps by drug-likeness. Molecular weight, hydrophobicity and polarity, reveals they preferentially occupy a relatively narrow range of possible values. According to this concept, all the compounds that fall within this range are described as "druglike". Of course this term does not define any obvious structural similarity to an approved drug. Druglikeness describe the impact of physicochemical properties in the molecular behavior *in vivo*.

Thus, we used the online software versions of Molinspiration software version 2016.10 (www.molinspiration.com) [20] in order to obtain the chemical structures and simplified molecular-input line-entry system (SMILES) notations of the synthesized nitrones. According to Lipinski's rule, the poor absorption or permeation is related to the presence of more than 5 H-bond donors and 10 H-bond acceptors. Furthermore, values of the molecular weight (MW) > 500 and calculated log P value > 5 lead to poor absorption/ permeability. We noticed that all Nitrones presented low lipophilicity values and MWs (Table 2). The bioavailability of bioactive molecules is characterized by their TPSA. This descriptor is highly correlated with the hydrogen bonding properties of a molecule. The TPSA values were observed in the range of 28.75–57.51 Å2. These values were lower the limit of 160 Å2 underlying a good oral bioavailability. The upper limit for the TPSA for a molecule to penetrate the brain is around 90 Å2. Thus, all these molecules are able to penetrate the brain (Table S1).

Table S1. In silico calculated physicochemical properties of the synthesized molecules, using the Molinspiration platform.

Nitrone	miLogP[a]	TPSA[b]	Natoms[c]	MW[d]	nON[e]	nOHNH[f]	Nviol[g]	rotb[h]	MV[i]
**PBN**	1.39	28.75	13	177.25	2	0	0	2	182.00
**HBN6**	1.46	57.51	24	316.36	4	0	0	4	290.39
**hBN1**	-0.34	57.51	17	234.30	4	0	0	3	230.33
**hBN2**	-0.04	57.51	19	254.29	4	0	0	3	235.55
**hBN3**	1.15	57.51	22	296.37	4	0	0	4	285.17
**hBN4**	-0.34	57.51	17	234.30	4	0	0	3	230.33
**hBN5**	-0.06	57.51	19	254.29	4	0	0	3	235.55
**hBN6**	1.15	57.51	22	296.37	4	0	0	4	285.17
**hBN7**	-0.39	57.51	17	234.30	4	0	0	3	230.33
**hBN8**	-0.09	57.51	19	254.29	4	0	0	3	235.55
**hBN9**	1.15	57.51	22	296.37	4	0	0	4	285.17

[a]Lipophilicity; [b] Topological Polar Surface Area; [*c]Number of atoms; [d]Molecular Weight; [e]Number of oxygen and nitrogen atoms; [f]Number of OH and NH groups; [g]Number of violations; [h]Number of rotatable bonds; [I] Molecular Volume.

2. Spectra of hBNs 1-9

hBN1

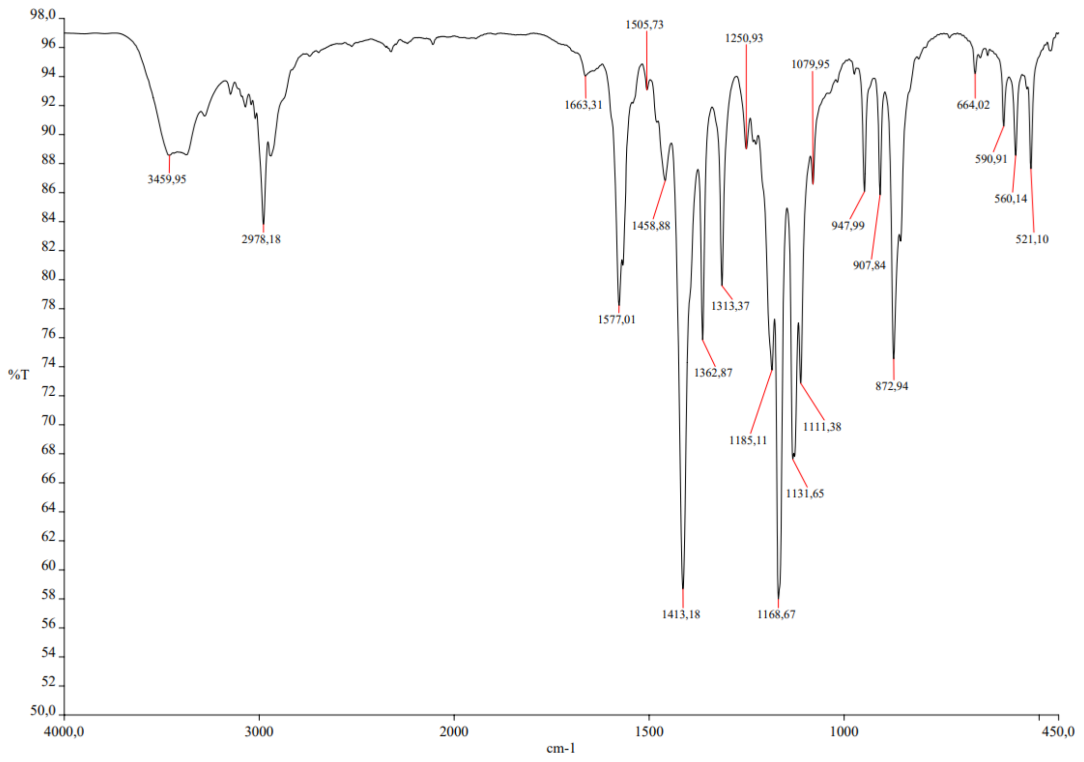

IR spectrum of hBN1

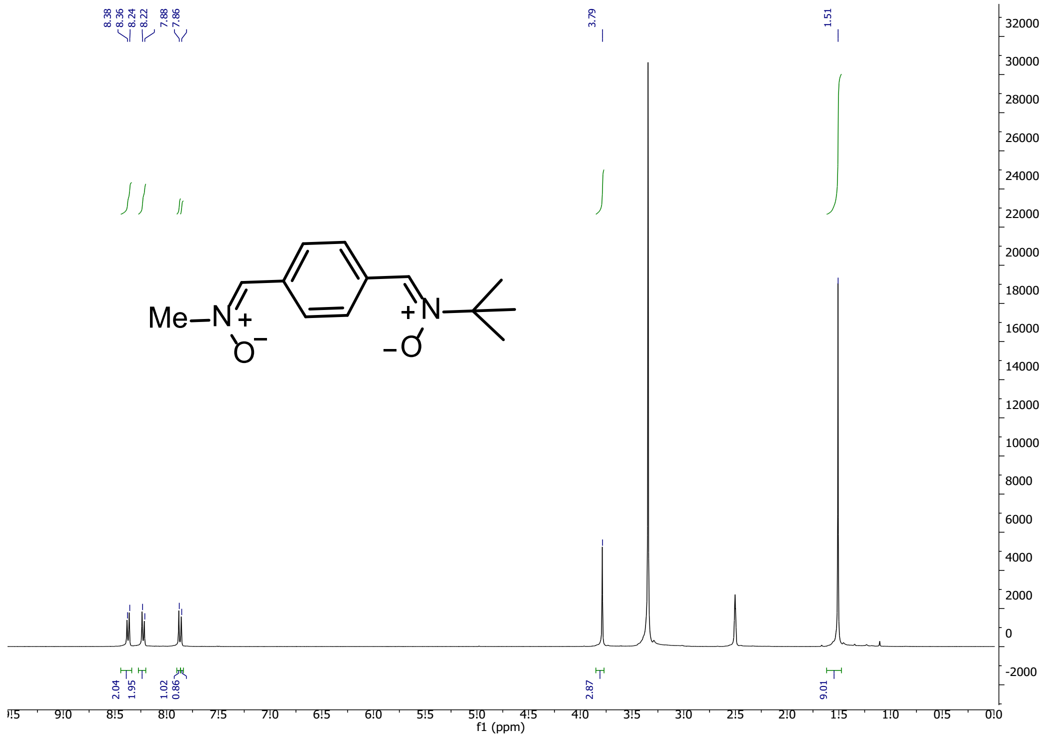

^{1}H spectrum of hBN1

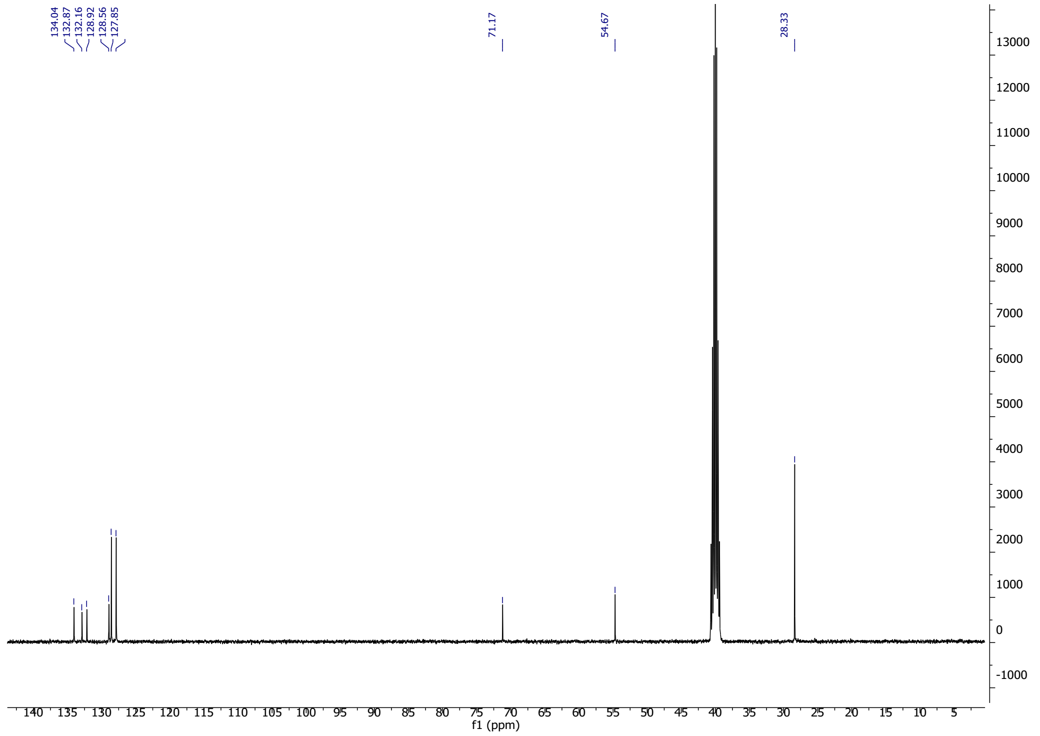

^{13}C NMR spectrum of hBN1

Qualitative Compound Report

**Data File**	1680_DDI-30_01.d	**Sample Name**	DDI-30
**Sample Type**	Sample	**Position**	Vial 2
**Instrument Name**	Instrument 1	**User Name**	
**Acq Method**	ESI_ACN_75_pos_new.m	**Acquired Time**	8/2/2022 11:26:36 AM (UTC+02:00)
**IRM Calibration Status**	Success	**DA Method**	Defecto_modificado.m
**Comment**			

**Sample Group**		**Info.**	
**User**	MIREIA TOLEDANO	**Stream Name**	LC 1
**Acquisition Time (Local)**	8/2/2022 11:26:36 AM (UTC+02:00)	**Acquisition SW Version**	6200 series TOF/6500 series Q-TOF B.08.00 (B8058.3 SP1)
**QTOF Driver Version**	8.00.00	**QTOF Firmware Version**	2.712
**Tune Mass Range Max.**	1700		

Compound Table

Compound Label	RT	Mass	Abund	Formula	Tgt Mass	Diff (ppm)	Hits (DB)
Cpd 1: C13 H18 N2 O2; 0.263	0.263	234.1369	147103	C13 H18 N2 O2	234.1368	0.52	1

Compound Label	*m/z*	RT	Algorithm	Mass
Cpd 1: C13 H18 N2 O2; 0.263	235.1439	0.263	Find by Formula	234.1369

MS Zoomed Spectrum

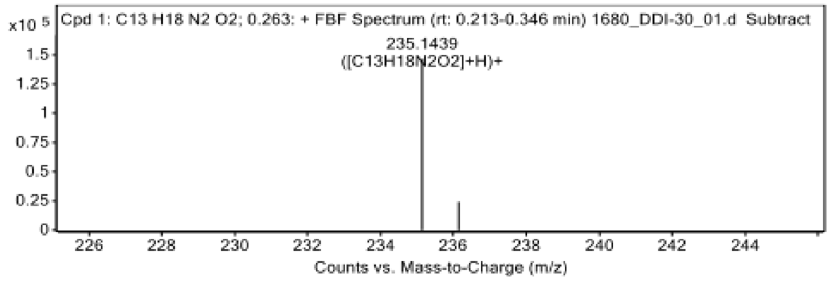

MS Spectrum Peak List

m/z	z	Abund	Formula	Ion
235.1439	1	147103.09	C13H18N2O2	(M+H)+
236.1492	1	24292.84	C13H18N2O2	(M+H)+

MS Zoomed Spectrum

Qualitative Compound Report

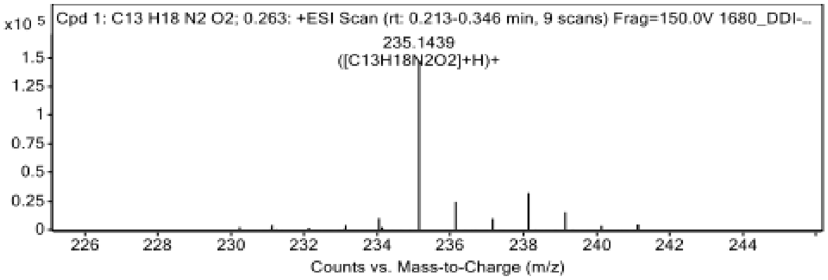

MS Spectrum Peak List

m/z	*Calc m/z*	Diff(ppm)	z	Abund	Formula	Ion
235.1439	235.1441	-0.81	1	147103.09	C13H18N2O2	(M+H)+
236.1492	236.1472	8.49	1	24292.84	C13H18N2O2	(M+H)+

--- End Of Report ---

HRMS spectrum of hBN1

hBN2

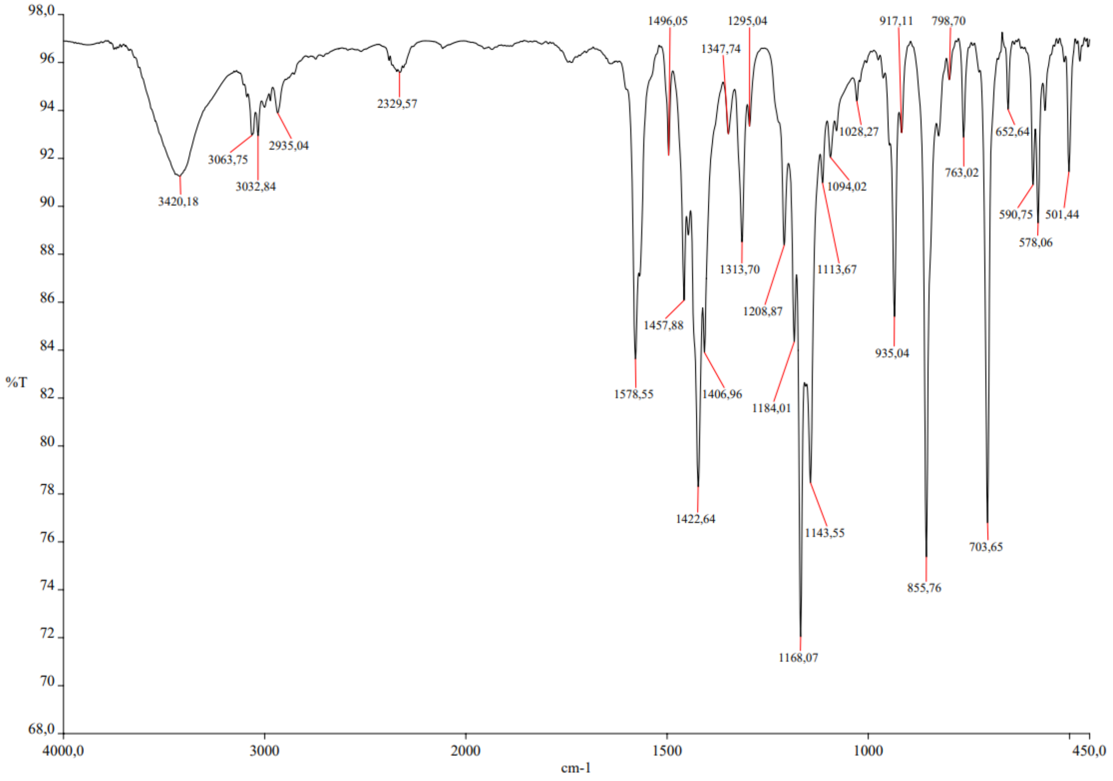

IR spectrum of hBN2

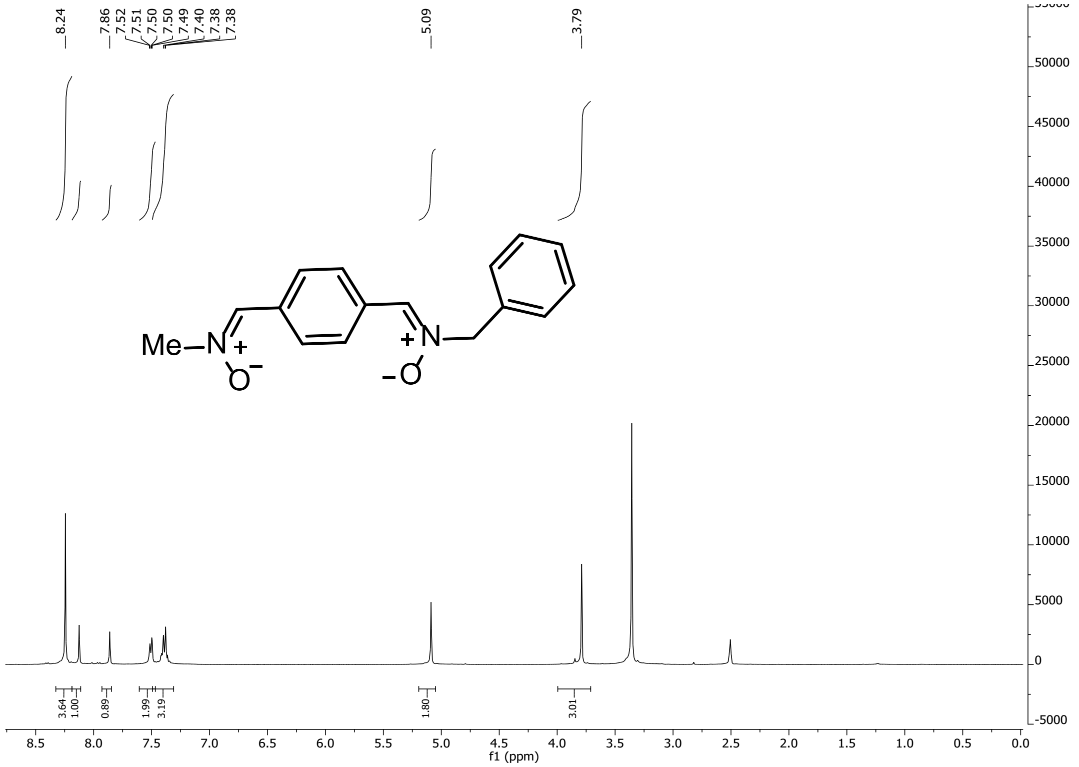

^{1}H NMR spectrum of hBN2

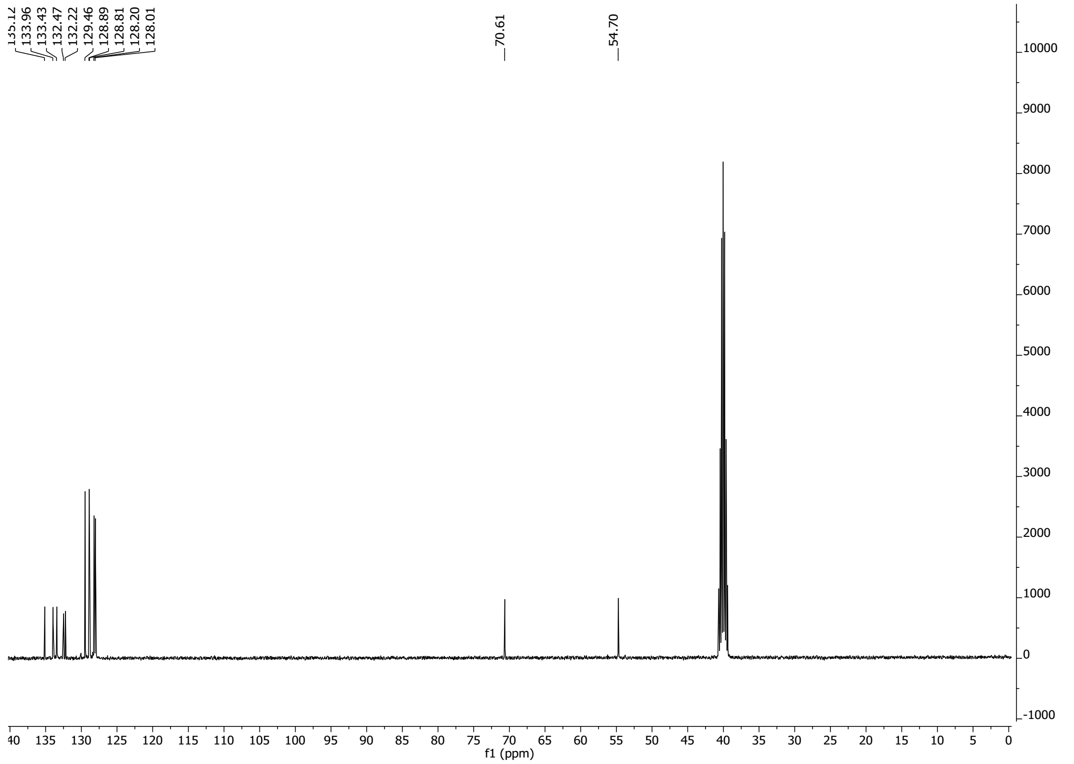

^{13}C NMR spectrum of hBN2

Qualitative Compound Report

**Data File**	1679_DDI-29_01.d	**Sample Name**	DDI-29
**Sample Type**	Sample	**Position**	Vial 1
**Instrument Name**	Instrument 1	**User Name**	
**Acq Method**	ESI_ACN_75_pos_new.m	**Acquired Time**	8/2/2022 11:19:22 AM (UTC+02:00)
**IRM Calibration Status**	Success	**DA Method**	Defecto_modificado.m
**Comment**			

**Sample Group**		**Info.**	
**User**	MIREIA TOLEDANO	**Stream Name**	LC 1
**Acquisition Time (Local)**	8/2/2022 11:19:22 AM (UTC+02:00)	**Acquisition SW Version**	6200 series TOF/6500 series Q-TOF B.08.00 (B8058.3 SP1)
**QTOF Driver Version**	8.00.00	**QTOF Firmware Version**	2.712
**Tune Mass Range Max.**	1700		

Compound Table

Compound Label	RT	Mass	Abund	Formula	Tgt Mass	Diff (ppm)	Hits (DB)
Cpd 1: C16 H16 N2 O2; 0.262	0.262	268.1205	9107147	C16 H16 N2 O2	268.1212	-2.53	1

Compound Label	*m/z*	RT	Algorithm	Mass
Cpd 1: C16 H16 N2 O2; 0.262	269.1278	0.262	Find by Formula	268.1205

MS Zoomed Spectrum

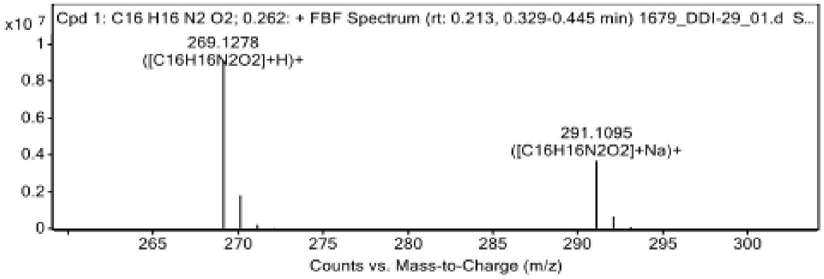

MS Spectrum Peak List

m/z	z	Abund	Formula	Ion
269.1278	1	9107147	C16H16N2O2	(M+H)+
270.1308	1	1797572.13	C16H16N2O2	(M+H)+
271.1345	1	199805.73	C16H16N2O2	(M+H)+
272.1392	1	19255.26	C16H16N2O2	(M+H)+
291.1095	1	3674424.5	C16H16N2O2	(M+Na)+
292.114	1	637951.5	C16H16N2O2	(M+Na)+
293.1171	1	78129.25	C16H16N2O2	(M+Na)+

294.1231	1	4466.18	C16H16N2O2	(M+Na)+

MS Zoomed Spectrum

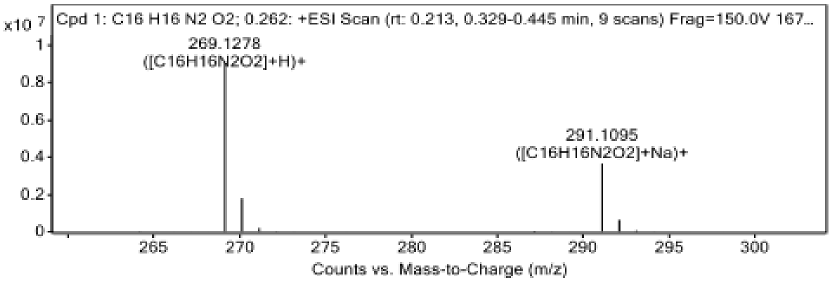

MS Spectrum Peak List

m/z	*Calc m/z*	Diff(ppm)	z	Abund	Formula	Ion
269.1278	269.1285	-2.53	1	9107147	C16H16N2O2	(M+H)+
270.1308	270.1316	-2.91	1	1797572.13	C16H16N2O2	(M+H)+
271.1345	271.1343	0.76	1	199805.73	C16H16N2O2	(M+H)+
272.1392	272.1369	8.52	1	19255.26	C16H16N2O2	(M+H)+
291.1095	291.1104	-3.19	1	3674424.5	C16H16N2O2	(M+Na)+
292.114	292.1135	1.72	1	637951.5	C16H16N2O2	(M+Na)+
293.1171	293.1162	3.03	1	78129.25	C16H16N2O2	(M+Na)+
294.1231	294.1188	14.41	1	4466.18	C16H16N2O2	(M+Na)+

--- End Of Report ---

HRMS spectrum of hBN2

hBN3

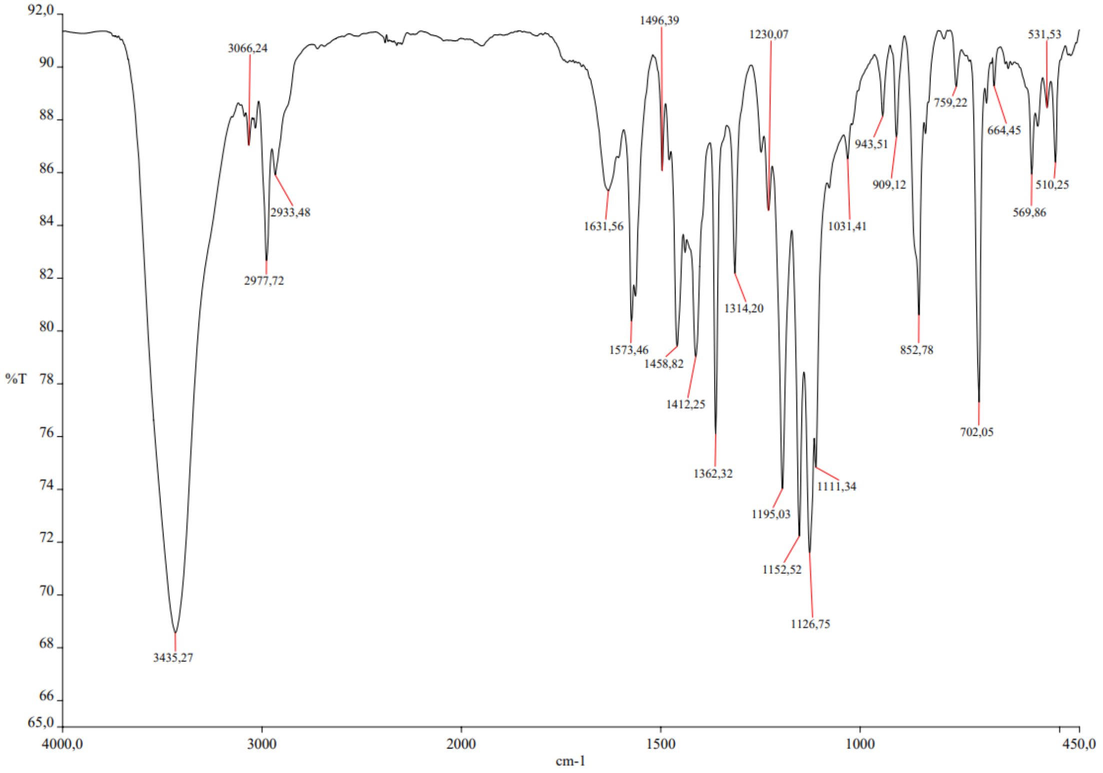

IR spectrum of hBN3

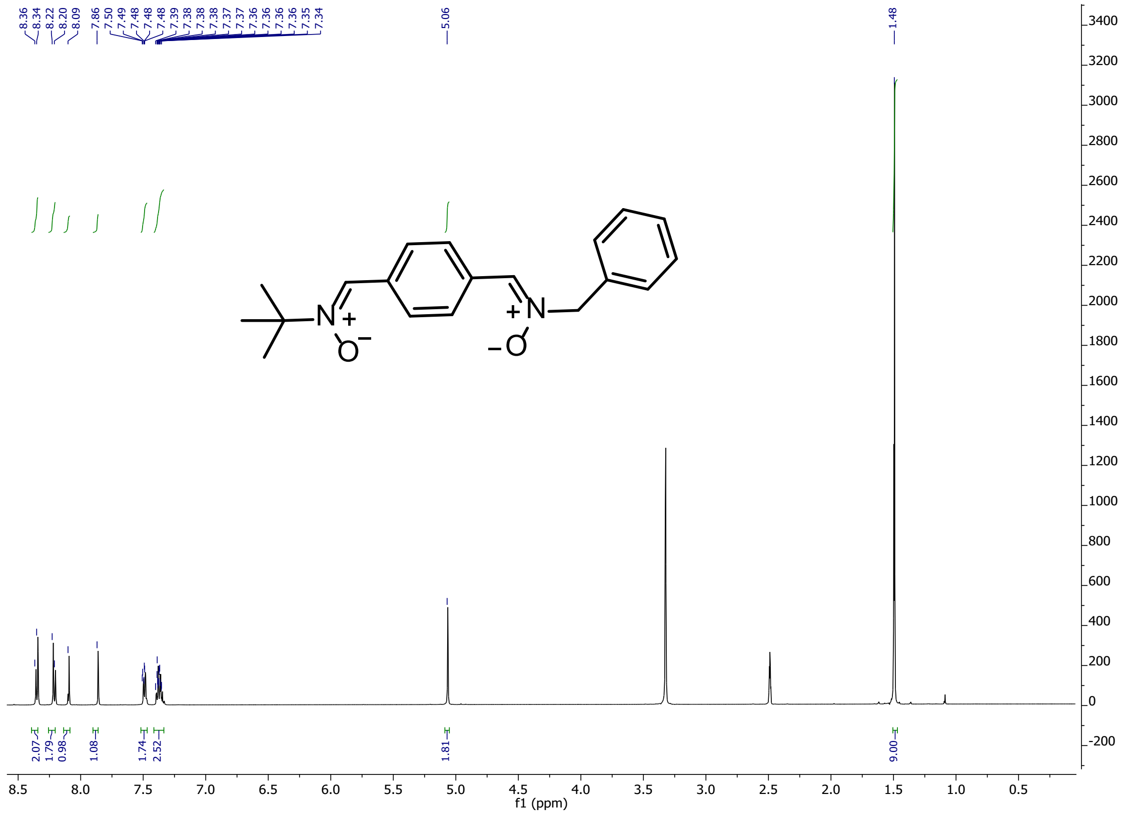

^{1}H NMR spectrum of hBN3

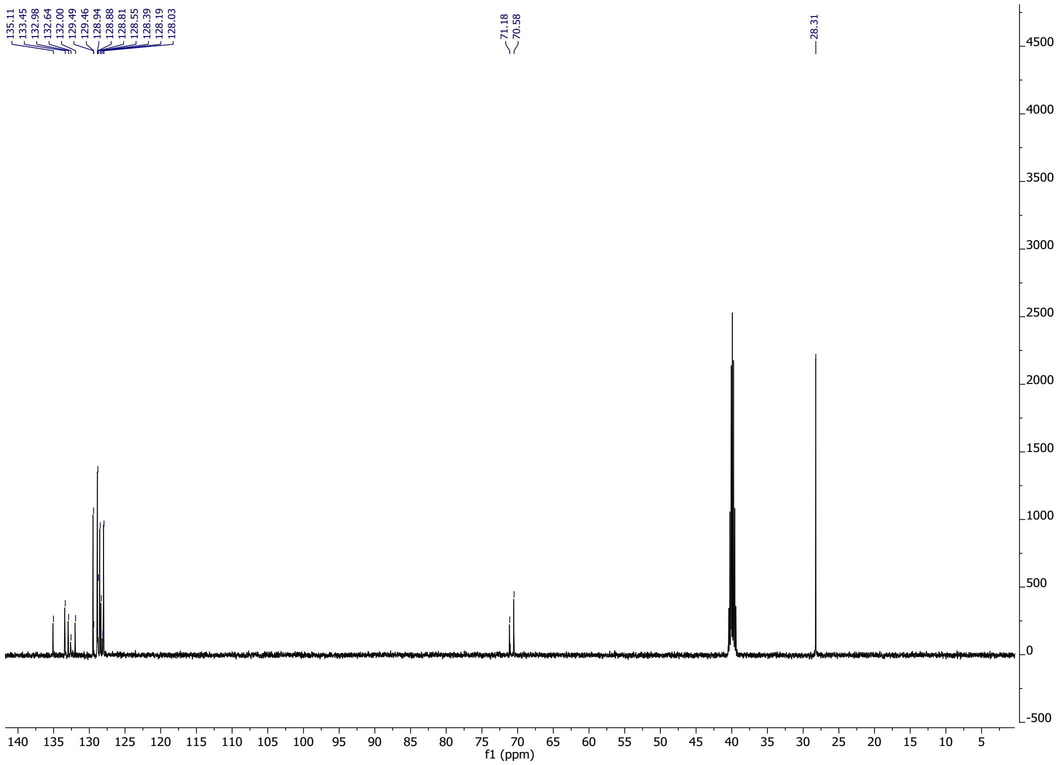

^{13}C NMR spectrum of hBN3

Qualitative Compound Report

**Data File**	1681_DDI-23_01.d	**Sample Name**	DDI-23
**Sample Type**	Sample	**Position**	Vial 3
**Instrument Name**	Instrument 1	**User Name**	
**Acq Method**	ESI_ACN_75_pos_new.m	**Acquired Time**	8/2/2022 11:33:53 AM (UTC+02:00)
**IRM Calibration Status**	Success	**DA Method**	Defecto_modificado.m
**Comment**			

**Sample Group**		**Info.**	
**User**	MIREIA TOLEDANO	**Stream Name**	LC 1
**Acquisition Time (Local)**	8/2/2022 11:33:53 AM (UTC+02:00)	**Acquisition SW Version**	6200 series TOF/6500 series Q-TOF B.08.00 (B8058.3 SP1)
**QTOF Driver Version**	8.00.00	**QTOF Firmware Version**	2.712
**Tune Mass Range Max.**	1700		

Compound Table

Compound Label	RT	Mass	Abund	Formula	Tgt Mass	Diff (ppm)	Hits (DB)
Cpd 1: C19 H22 N2 O2; 0.276	0.276	310.168	11299569	C19 H22 N2 O2	310.1681	-0.29	1

Compound Label	*m/z*	RT	Algorithm	Mass
Cpd 1: C19 H22 N2 O2; 0.276	311.1754	0.276	Find by Formula	310.168

MS Zoomed Spectrum

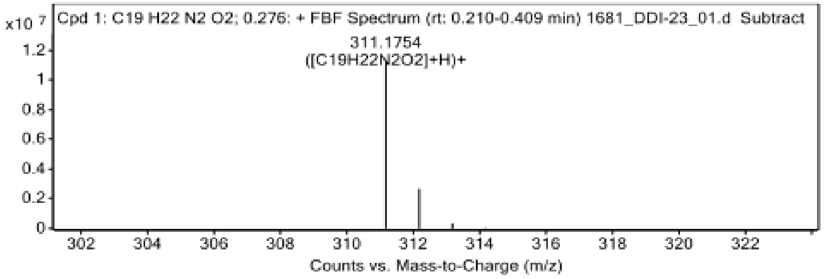

MS Spectrum Peak List

m/z	z	Abund	Formula	Ion
311.1754	1	11299569	C19H22N2O2	(M+H)+
312.1782	1	2675737	C19H22N2O2	(M+H)+
313.1823	1	326884.31	C19H22N2O2	(M+H)+
314.1853	1	27781.73	C19H22N2O2	(M+H)+

MS Zoomed Spectrum

Qualitative Compound Report

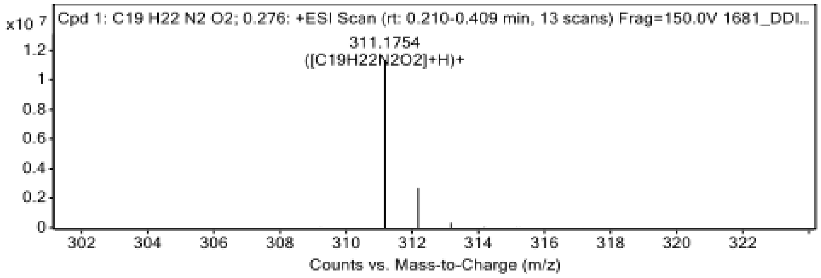

MS Spectrum Peak List

m/z	*Calc m/z*	Diff(ppm)	z	Abund	Formula	Ion
311.1754	311.1754	-0.16	1	11299569	C19H22N2O2	(M+H)+
312.1782	312.1786	-1.26	1	2675737	C19H22N2O2	(M+H)+
313.1823	313.1814	2.71	1	326884.31	C19H22N2O2	(M+H)+
314.1853	314.1841	3.7	1	27781.73	C19H22N2O2	(M+H)+

--- End Of Report ---

HRMS spectrum of hBN3

hBN4

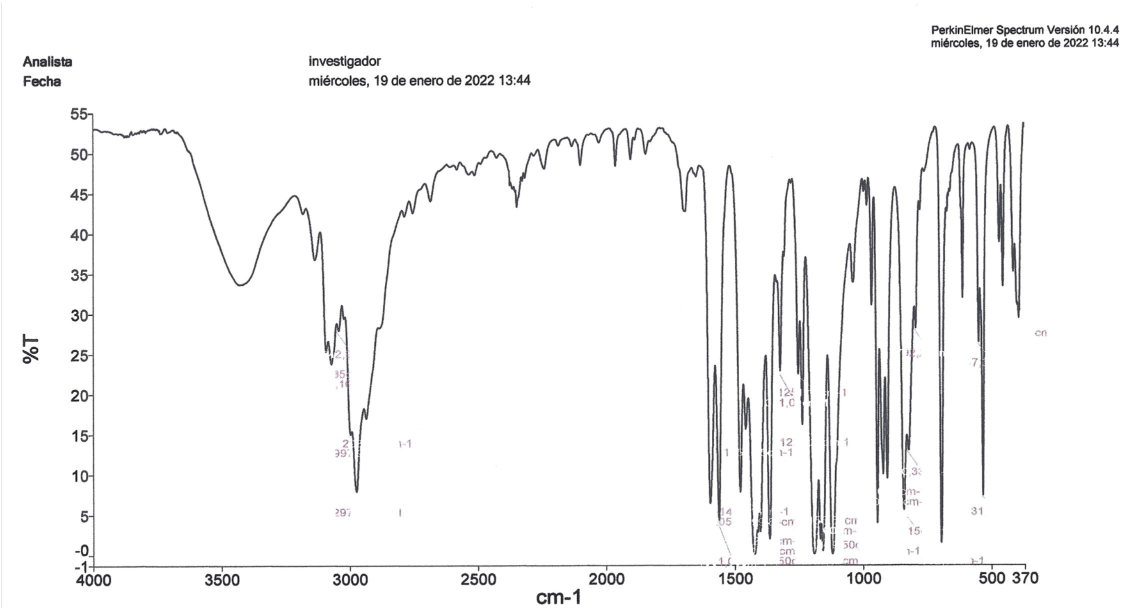

IR spectrum of hBN4

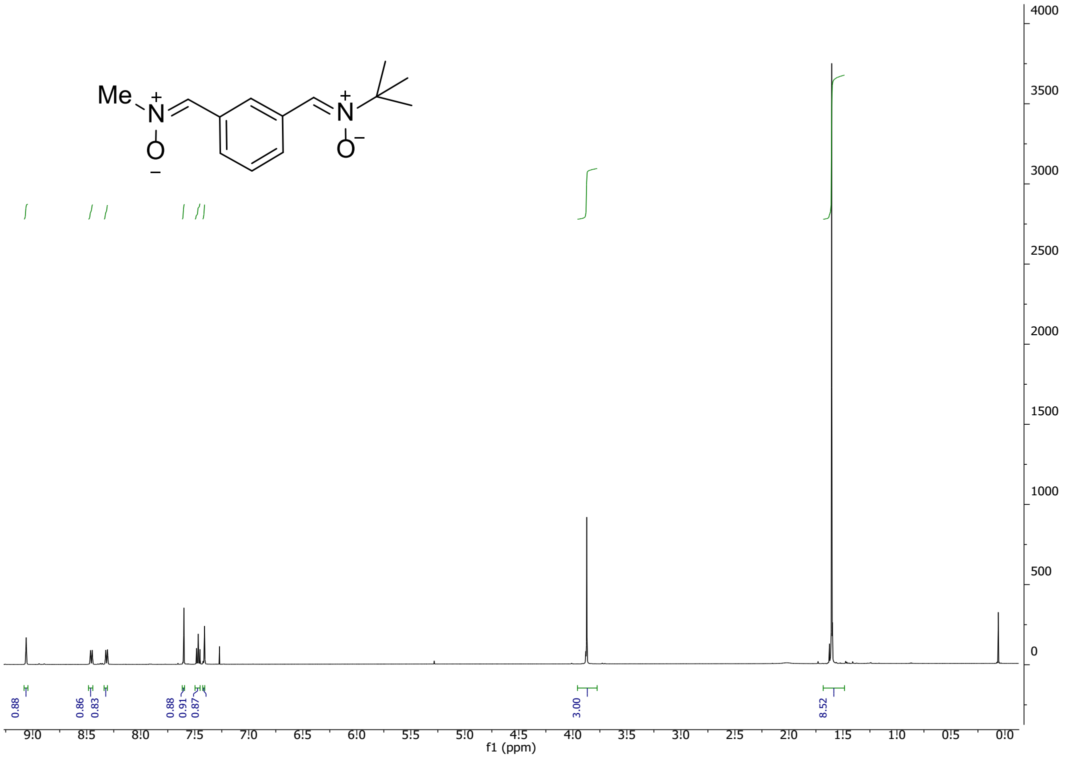

^{1}H NMR spectrum of hBN4

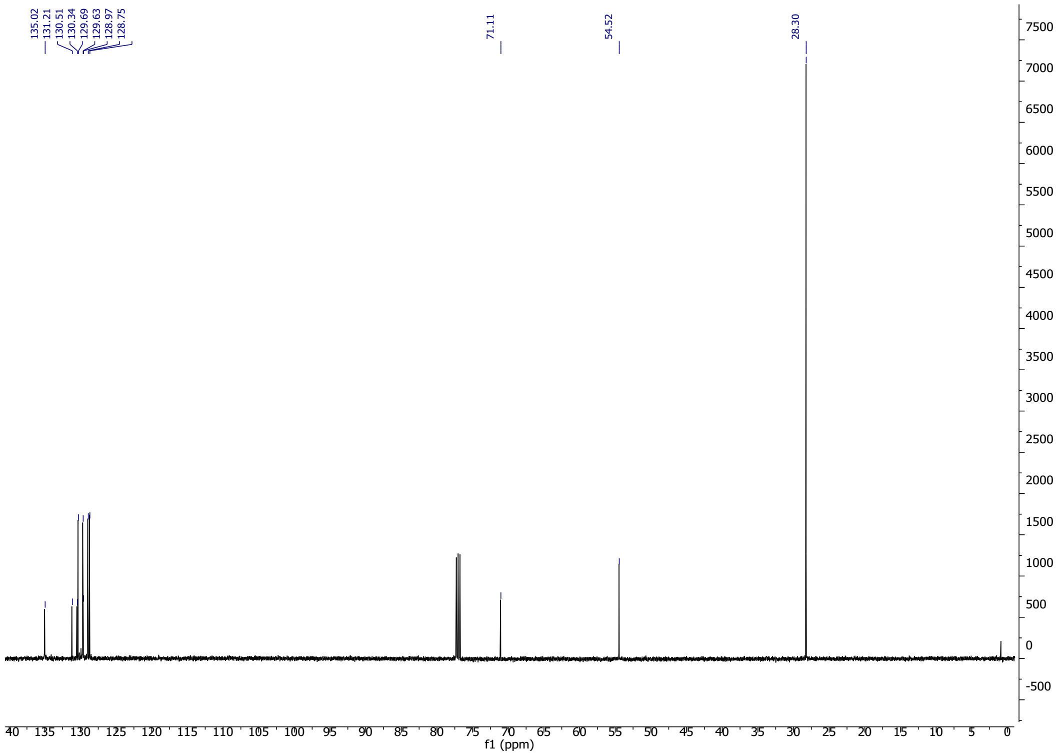

^{13}C NMR spectrum of hBN4

Qualitative Compound Report

**Data File**	14666_DDI_106_01.d	**Sample Name**	DDI_106
**Sample Type**	Sample	**Position**	Vial 5
**Instrument Name**	Instrument 1	**User Name**	
**Acq Method**	ESI_ACN_75_pos.m	**IRM Calibration Status**	Success
**DA Method**	Defecto_modificado_CS.m	**Comment**	

Compound Table

Compound Label	RT	Mass	Abund	Formula	Tgt Mass	Diff (ppm)
Cpd 1: C13 H18 N2 O2	0.971	234.1373	167579	C13 H18 N2 O2	234.13683	2.03

Compound Label	RT	Algorithm	Mass
Cpd 1: C13 H18 N2 O2	0.971	Find By Formula	234.1373

MS Zoomed Spectrum

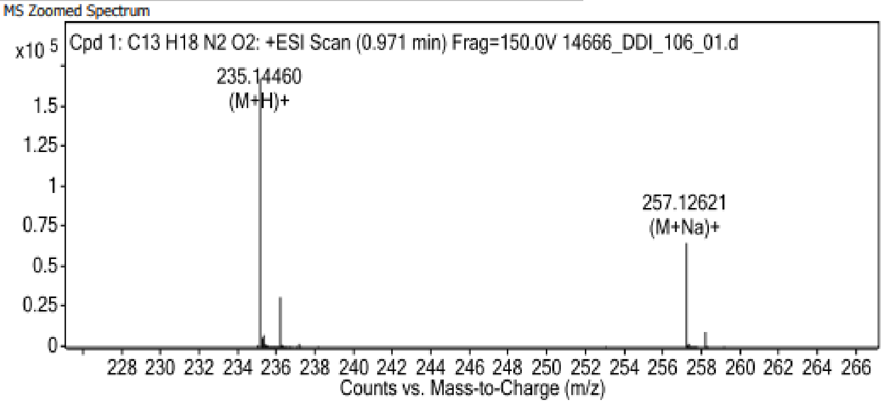

MS Spectrum Peak List

m/z	*Calc m/z*	Diff(ppm)	z	Abund	Formula	Ion
235.00249				1961		
235.1446	235.1441	2.09		167579	C13 H19 N2 O2	(M+H)+
235.22777				6624		
235.28619				7939		
235.33721				2606		
235.47971				1685		
236.14793	236.1472	3.07		31550	C13 H19 N2 O2	(M+H)+
237.14989	237.14974	0.64		3123	C13 H19 N2 O2	(M+H)+
257.12621	257.12605	0.61	1	65585	C13 H18 N2 Na O2	(M+Na)+
258.12806	258.12914	-4.19	1	10103	C13 H18 N2 Na O2	(M+Na)+

--- End Of Report ---

HRMS spectrum of hBN4

hBN5

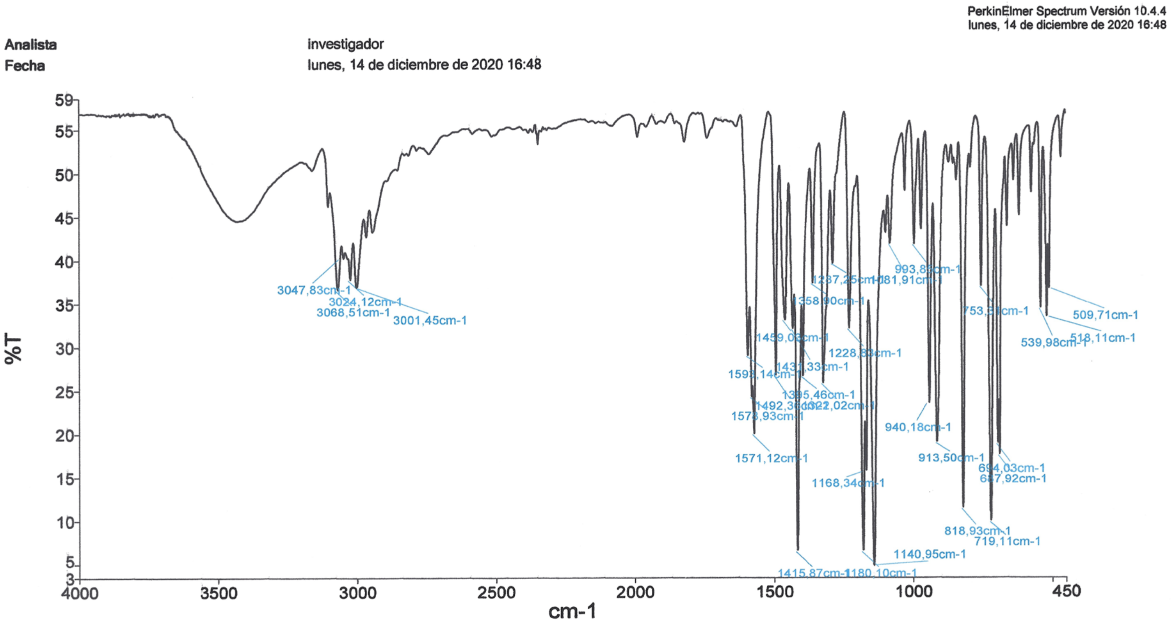

IR spectrum of hBN5

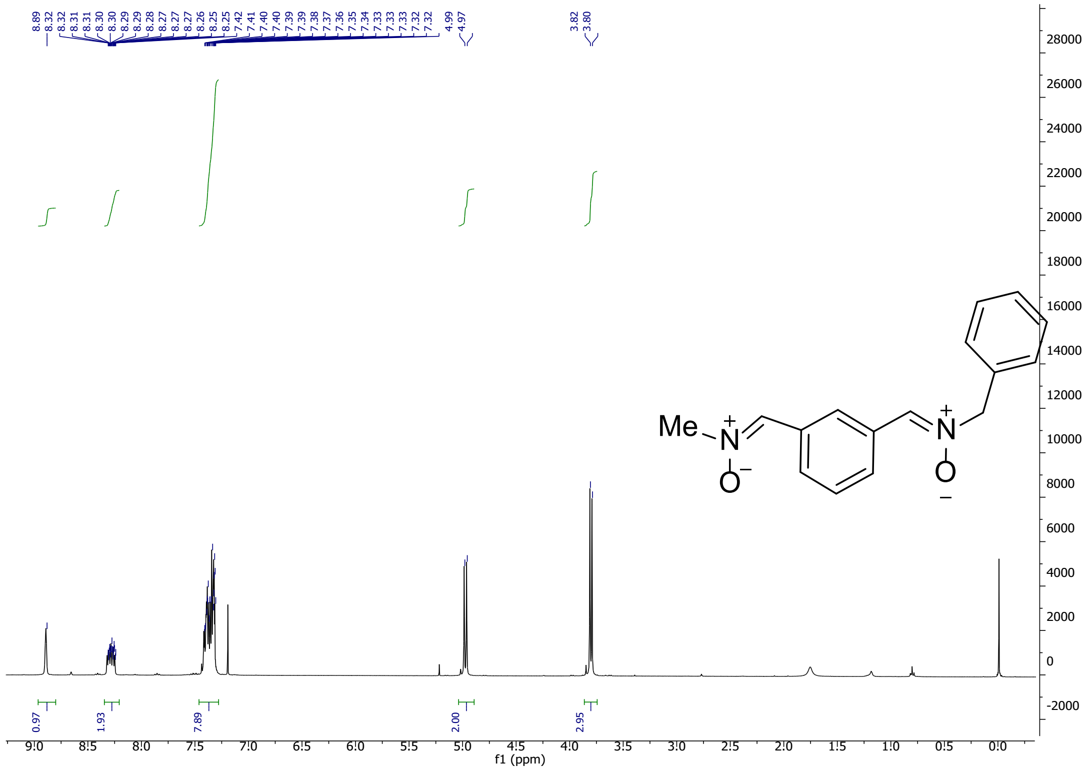

^{1}H NMR spectrum of hBN5

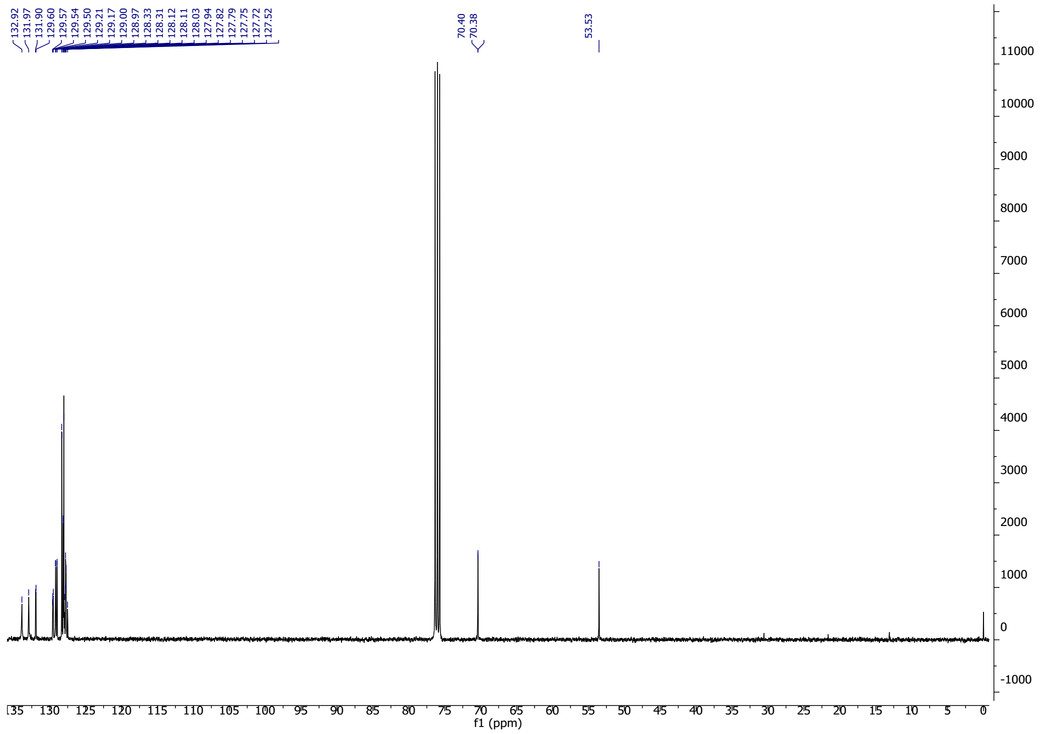

^{13}C NMR spectrum of hBN5

Qualitative Compound Report

**Data File**	13964_DDI_91_01.d	**Sample Name**	DDI_91
**Sample Type**	Sample	**Position**	Vial 31
**Instrument Name**	Instrument 1	**User Name**	
**Acq Method**	ESI_ACN_75_pos.m	**IRM Calibration Status**	Some Ions Missed
**DA Method**	Defecto_modificado_CS.m	**Comment**	

Compound Table

Compound Label	RT	Mass	Abund	Formula	Tgt Mass	Diff (ppm)
Cpd 1: C16 H16 N2 O2	0.471	268.12191	184475	C16 H16 N2 O2	268.12118	2.73

Compound Label	RT	Algorithm	Mass
Cpd 1: C16 H16 N2 O2	0.471	Find By Formula	268.12191

MS Zoomed Spectrum

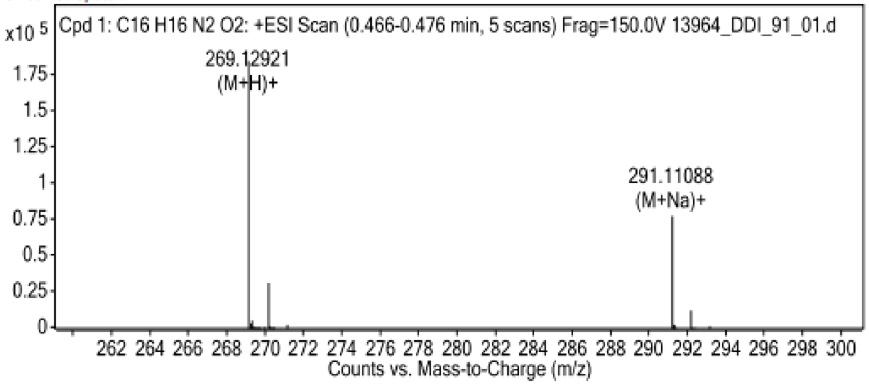

MS Spectrum Peak List

m/z	*Calc m/z*	Diff(ppm)	z	Abund	Formula	Ion
269.12921	269.12845	2.79		184475	C16 H17 N2 O2	(M+H)+
269.20896				3631		
269.25849				1578		
269.27979				6439		
270.13237	270.13159	2.9		31803	C16 H17 N2 O2	(M+H)+
270.1846				1634		
271.13429	271.1343	-0.02		3323	C16 H17 N2 O2	(M+H)+
291.11088	291.1104	1.64	1	77789	C16 H16 N2 Na O2	(M+Na)+
292.11398	292.11353	1.52	1	12969	C16 H16 N2 Na O2	(M+Na)+
293.11615	293.11624	-0.31	1	1619	C16 H16 N2 Na O2	(M+Na)+

--- End Of Report ---

HRMS spectrum of hBN5

hBN6

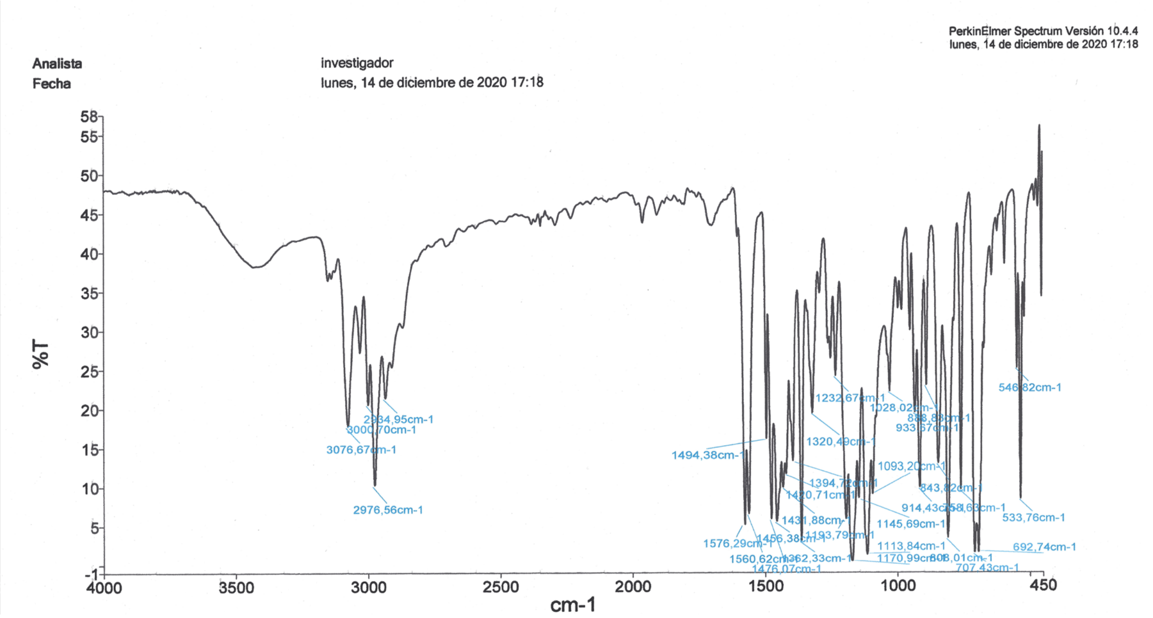

IR spectrum of hBN6

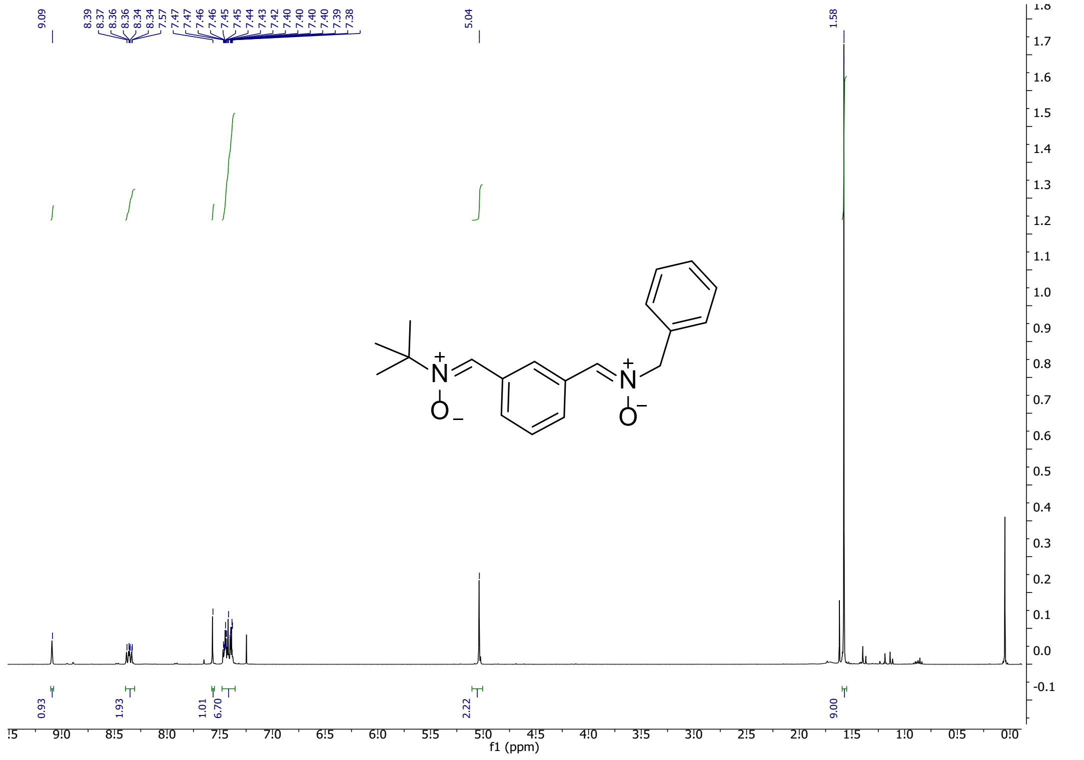

^{1}H NMR spectrum of hBN6

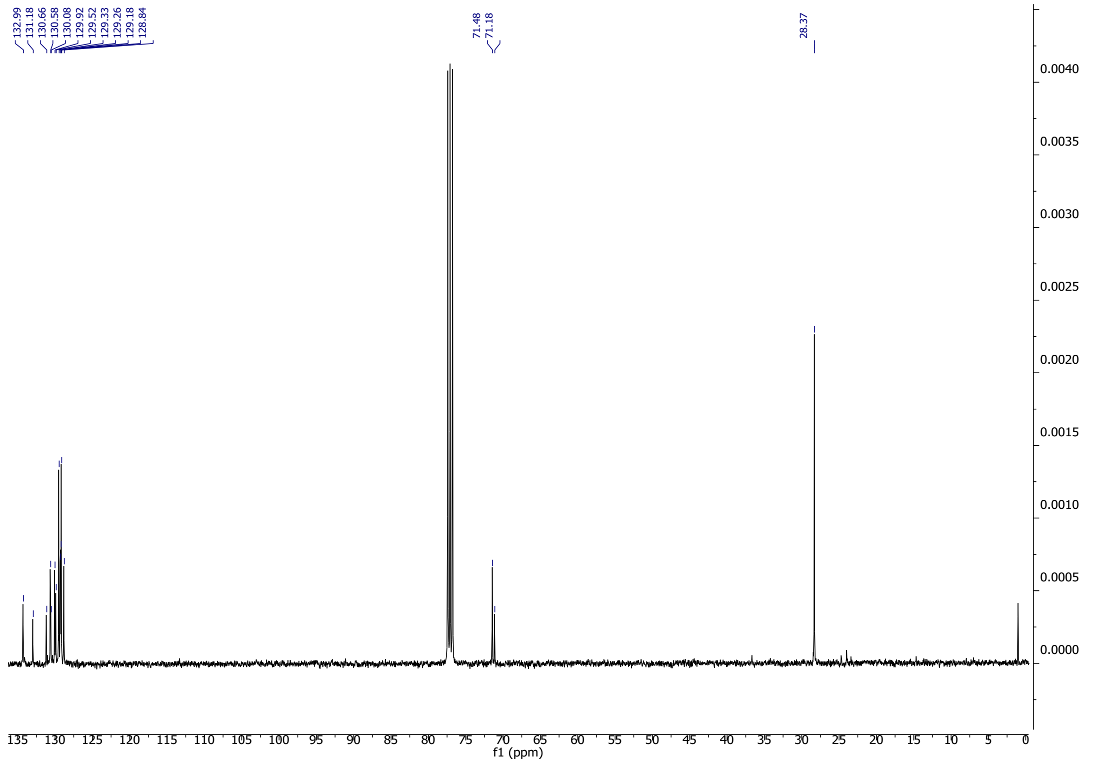

*

^{13}C NMR spectrum of hBN6

Qualitative Compound Report

**Data File**	13965_DDI_92_01.d	**Sample Name**	DDI_92
**Sample Type**	Sample	**Position**	Vial 32
**Instrument Name**	Instrument 1	**User Name**	
**Acq Method**	ESI_ACN_75_pos.m	**IRM Calibration Status**	Some Ions Missed
**DA Method**	Defecto_modificado_CS.m	**Comment**	

Compound Table

Compound Label	RT	Mass	Abund	Formula	Tgt Mass	Diff (ppm)
Cpd 1: C19 H22 N2 O2	0.799	310.16899	95067	C19 H22 N2 O2	310.16813	2.77

Compound Label	RT	Algorithm	Mass
Cpd 1: C19 H22 N2 O2	0.799	Find By Formula	310.16899

MS Zoomed Spectrum

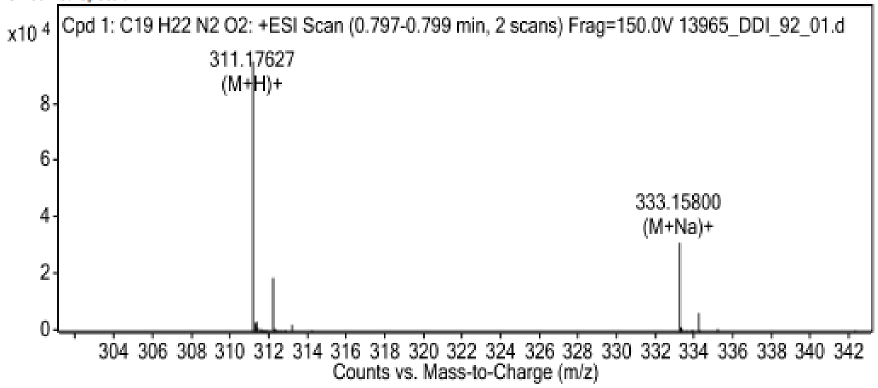

MS Spectrum Peak List

m/z	*Calc m/z*	Diff(ppm)	z	Abund	Formula	Ion
311.17627	311.1754	2.78		95067	C19 H23 N2 O2	(M+H)+
311.24588				3064		
311.29871				1013		
311.33784				3474		
311.41279				1345		
312.17966	312.17858	3.43		18892	C19 H23 N2 O2	(M+H)+
312.2375				1199		
313.18109	313.18143	-1.09		2374	C19 H23 N2 O2	(M+H)+
333.158	333.15735	1.94	1	31477	C19 H22 N2 Na O2	(M+Na)+
334.16054	334.16053	0.04	1	6447	C19 H22 N2 Na O2	(M+Na)+

--- End Of Report ---

HRMS spectrum of hBN6

hBN7

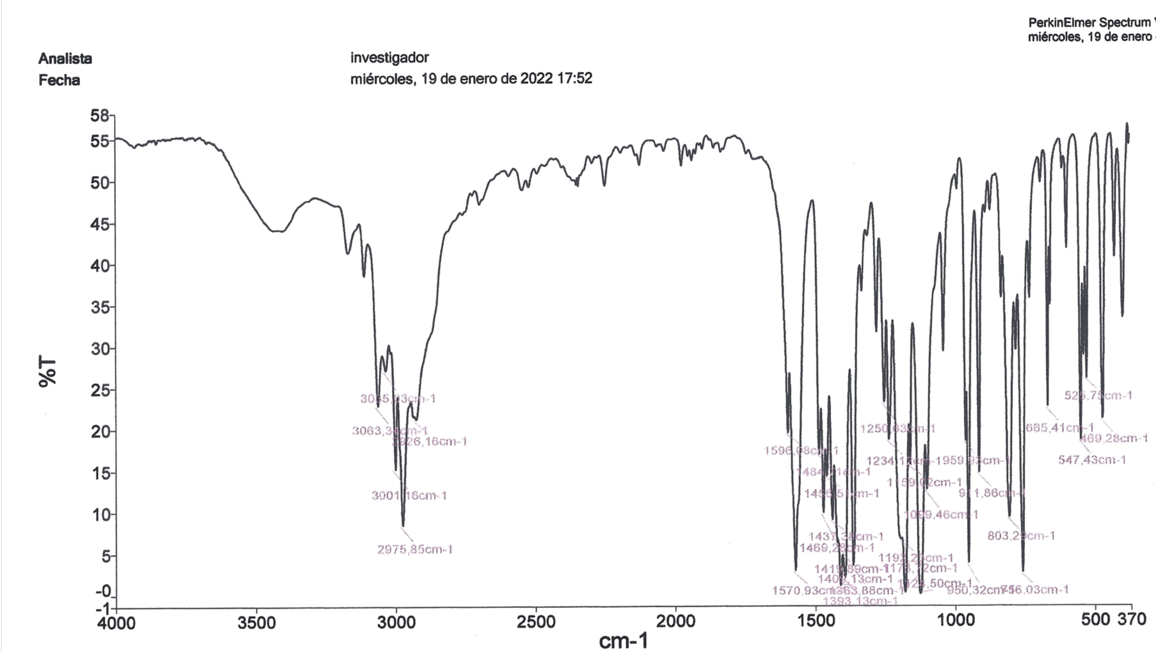

IR spectrum of hBN7

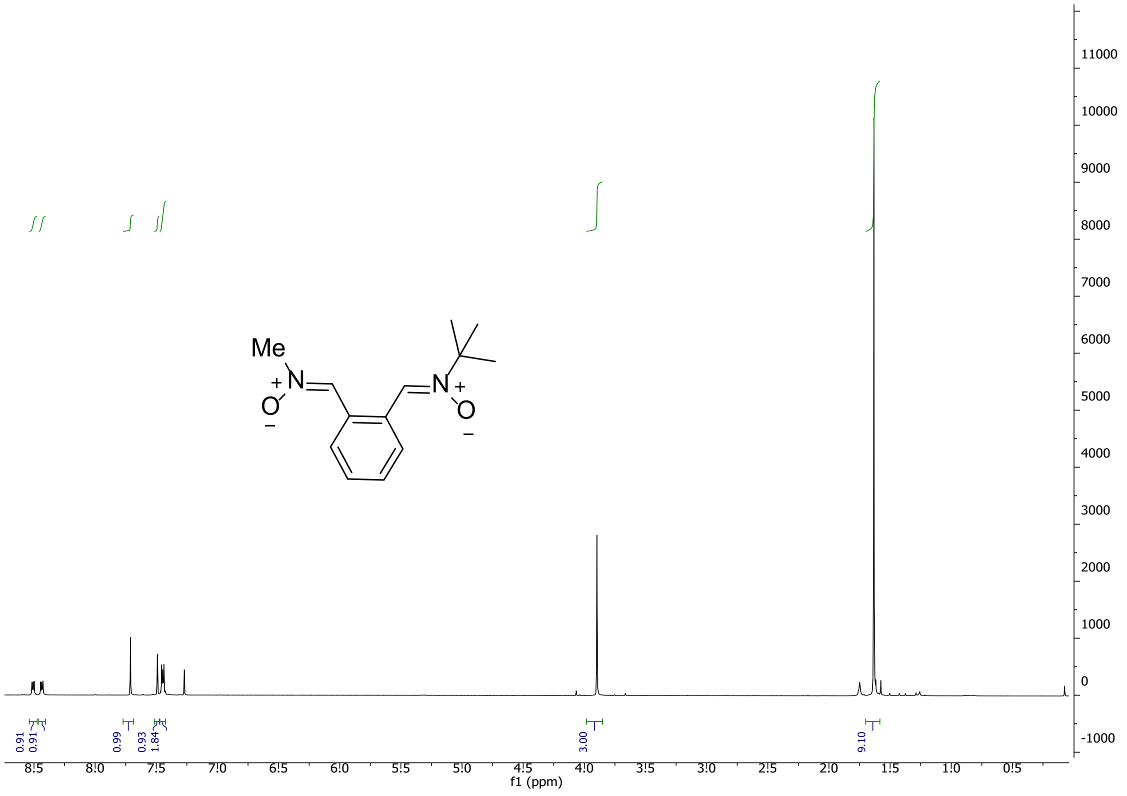

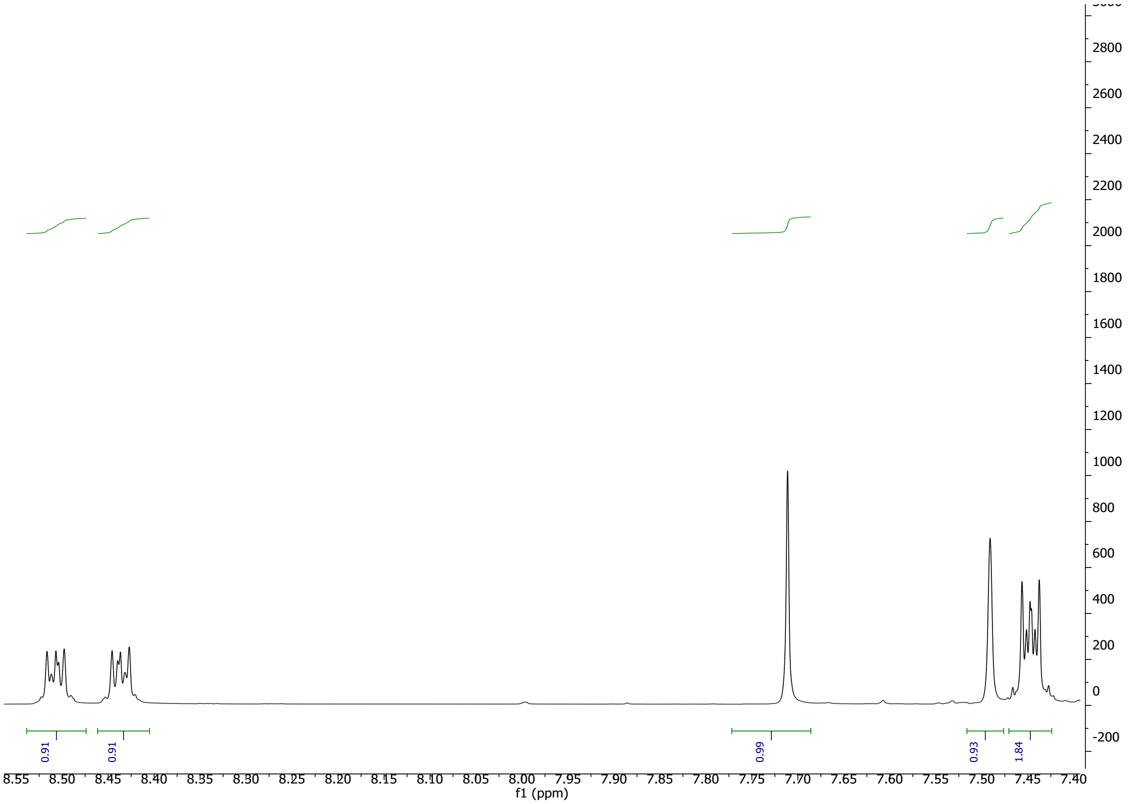

^{1}H NMR spectrum of hBN7

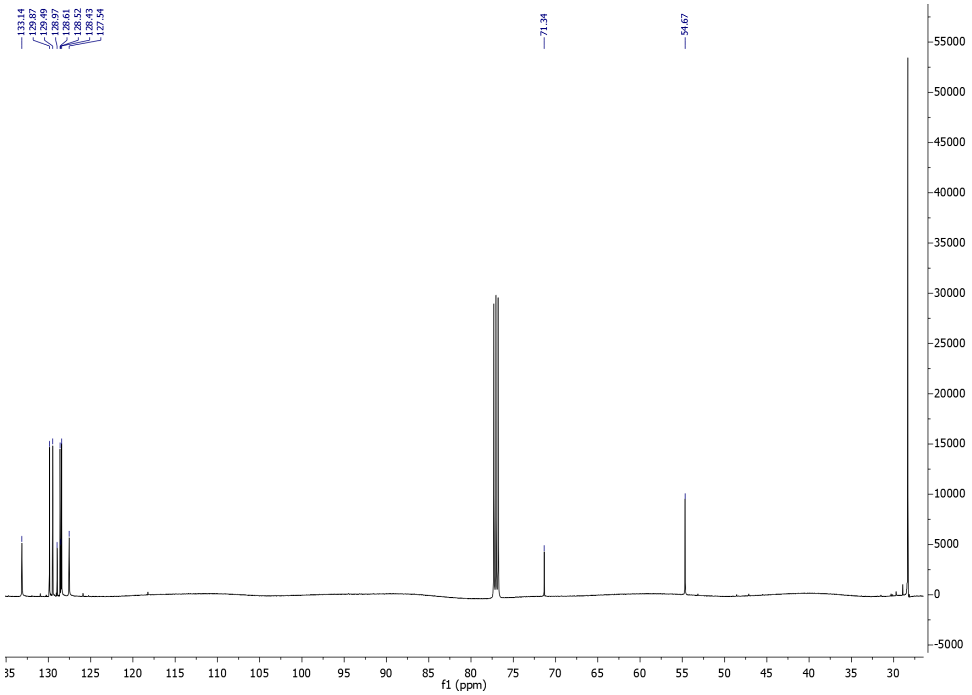

^{13}C NMR spectrum of hBN7

Qualitative Compound Report

**Data File**	14669_DDI_109_01.d	**Sample Name**	DDI_109
**Sample Type**	Sample	**Position**	Vial 8
**Instrument Name**	Instrument 1	**User Name**	
**Acq Method**	ESI_ACN_75_pos.m	**IRM Calibration Status**	Success
**DA Method**	Defecto_modificado_CS.m	**Comment**	

Compound Table

Compound Label	RT	Mass	Abund	Formula	Tgt Mass	Diff (ppm)
Cpd 1: C13 H18 N2 O2	1.533	234.13682	134788	C13 H18 N2 O2	234.13683	-0.02

Compound Label	RT	Algorithm	Mass
Cpd 1: C13 H18 N2 O2	1.533	Find By Formula	234.13682

MS Zoomed Spectrum

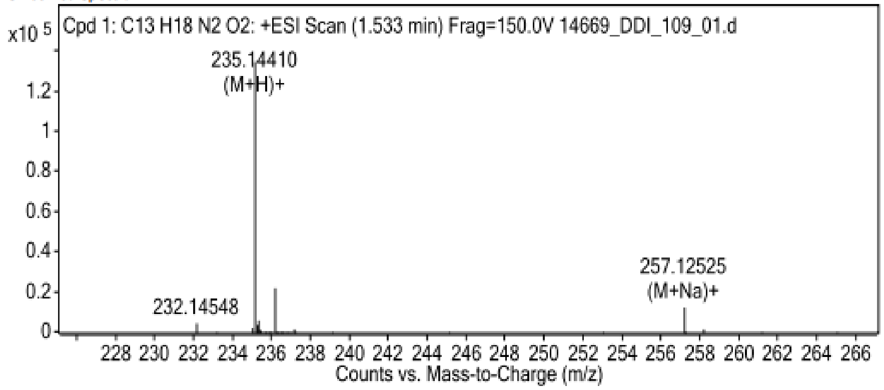

MS Spectrum Peak List

m/z	*Calc m/z*	Diff(ppm)	z	Abund	Formula	Ion
232.14548				5356		
234.99905				3211		
235.1441	235.1441	-0.02		134788	C13 H19 N2 O2	(M+H)+
235.22723				4331		
235.28574				6391		
235.3409				1881		
236.14735	236.1472	0.65		22643	C13 H19 N2 O2	(M+H)+
237.1508	237.14974	4.5		2500	C13 H19 N2 O2	(M+H)+
257.12525	257.12605	-3.12	1	13216	C13 H18 N2 Na O2	(M+Na)+
258.13029	258.12914	4.45	1	2032	C13 H18 N2 Na O2	(M+Na)+

--- End Of Report ---

HRMS spectrum of hBN7

hBN8

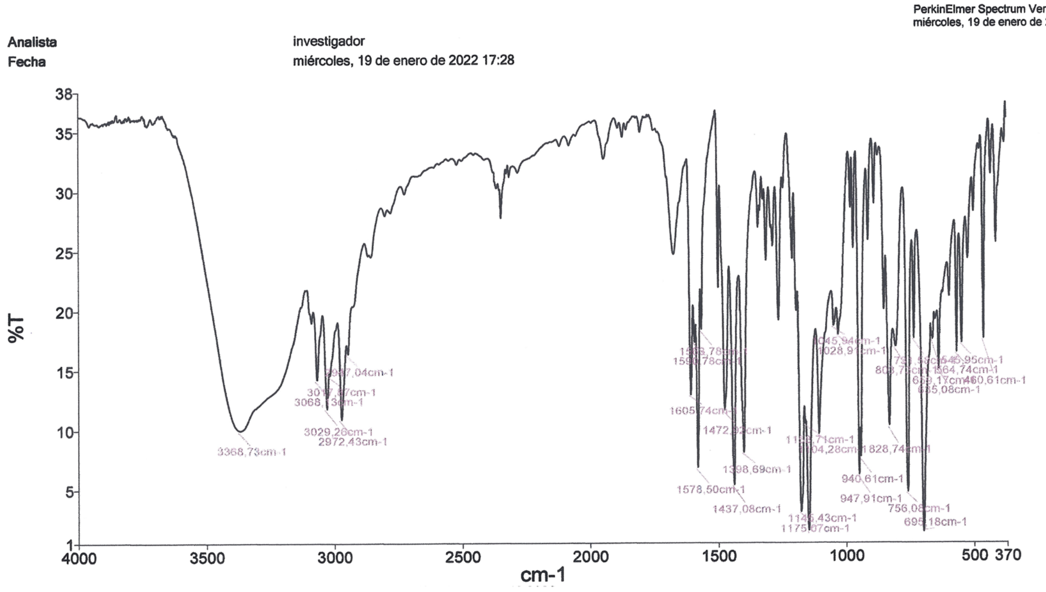

IR spectrum of hBN8

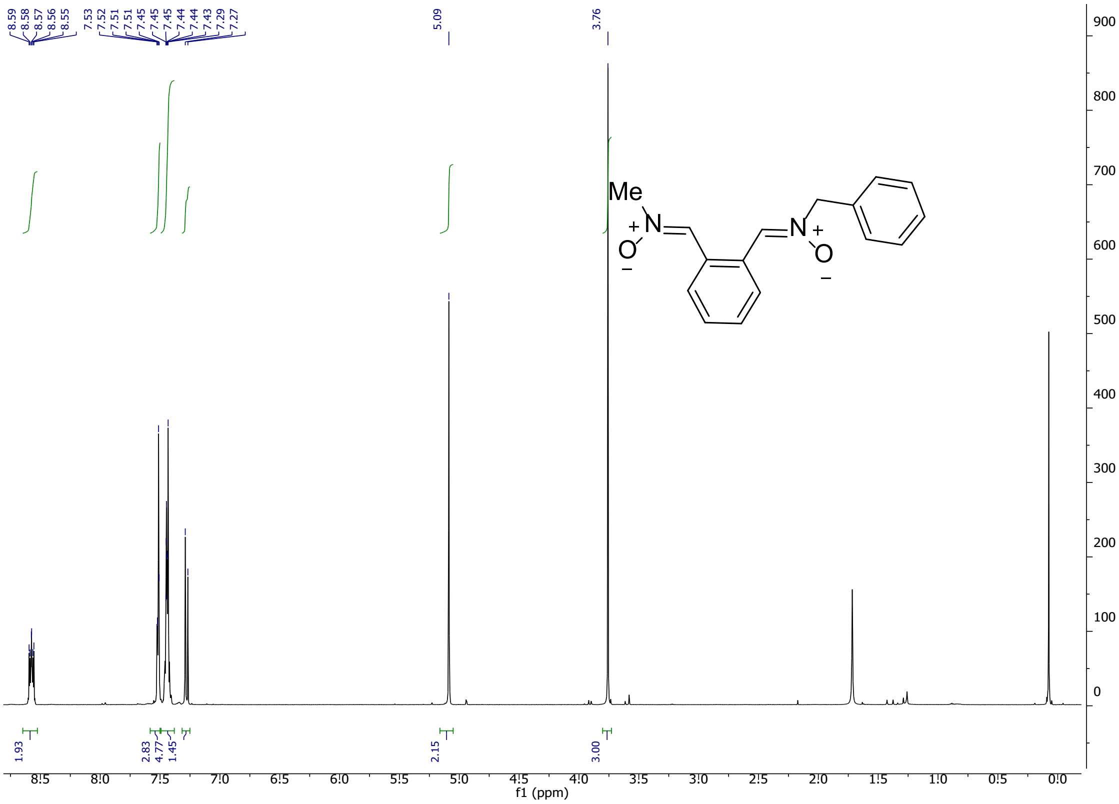

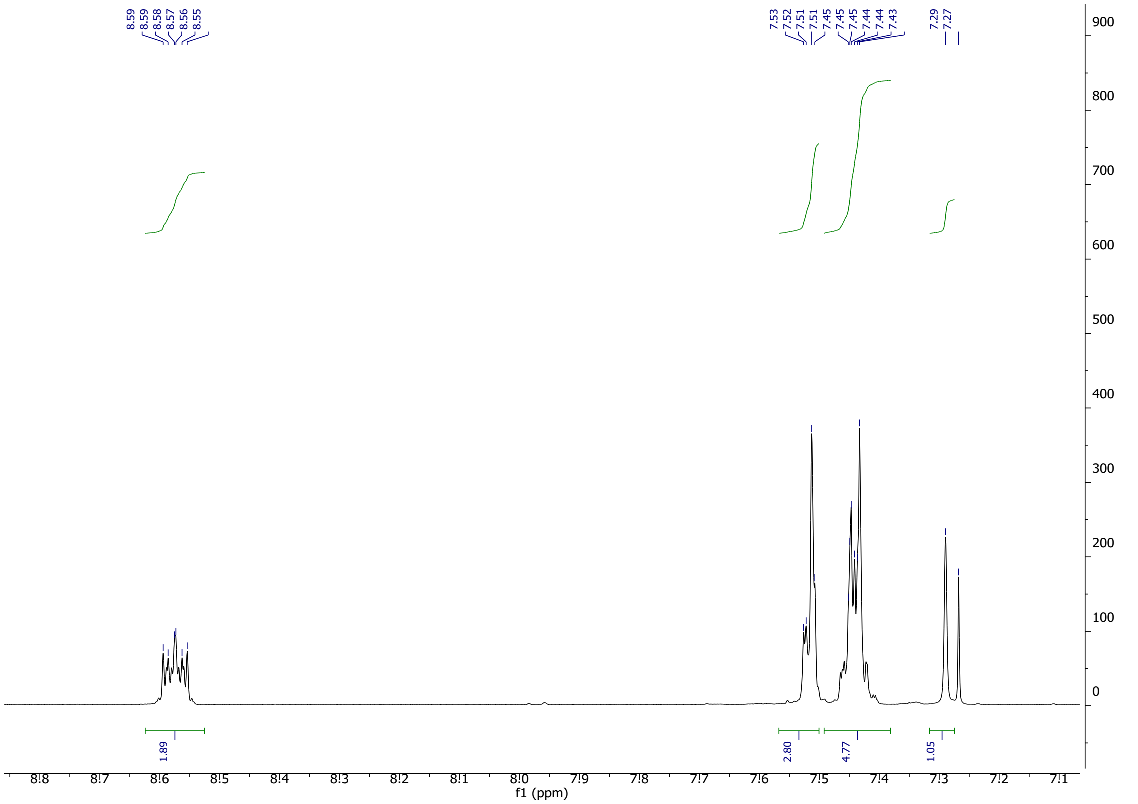

^{1}H NMR spectrum of hBN8

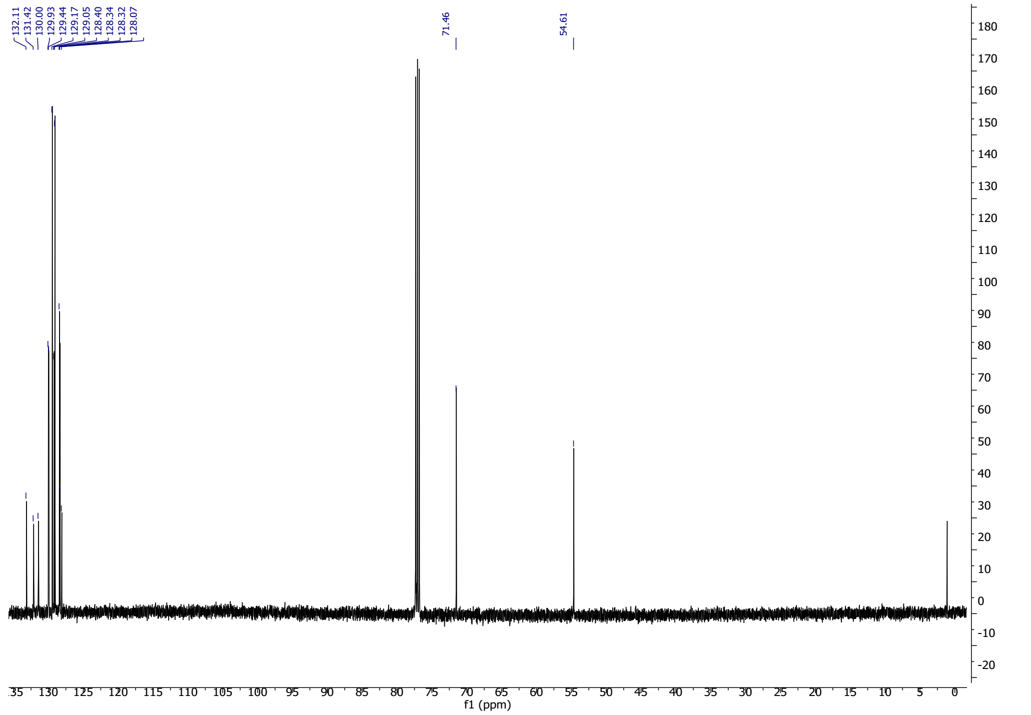

^{13}C NMR spectra of hBN8

Qualitative Compound Report

**Data File**	14668_DDI_108_01.d	**Sample Name**	DDI_108
**Sample Type**	Sample	**Position**	Vial 7
**Instrument Name**	Instrument 1	**User Name**	
**Acq Method**	ESI_ACN_75_pos.m	**IRM Calibration Status**	Success
**DA Method**	Defecto_modificado_CS.m	**Comment**	

Compound Table

Compound Label	RT	Mass	Abund	Formula	Tgt Mass	Diff (ppm)
Cpd 1: C16 H16 N2 O2	1.007	268.12174	164456	C16 H16 N2 O2	268.12118	2.1

Compound Label	RT	Algorithm	Mass
Cpd 1: C16 H16 N2 O2	1.007	Find By Formula	268.12174

MS Zoomed Spectrum

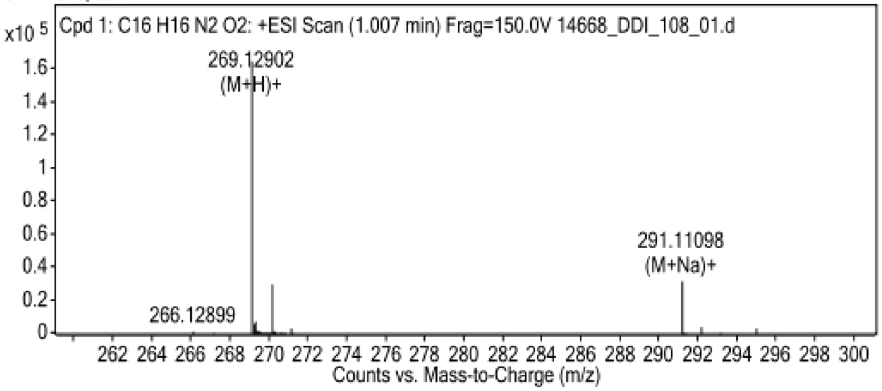

MS Spectrum Peak List

m/z	*Calc m/z*	Diff(ppm)	z	Abund	Formula	Ion
266.12899				2071		
269.12902	269.12845	2.09		164456	C16 H17 N2 O2	(M+H)+
269.21974				6127		
269.28054				8007		
269.33557				2319		
269.47521				1741		
270.13211	270.13159	1.9		30356	C16 H17 N2 O2	(M+H)+
271.13552	271.1343	4.49		3328	C16 H17 N2 O2	(M+H)+
291.11098	291.1104	2.01	1	32081	C16 H16 N2 Na O2	(M+Na)+
292.11424	292.11353	2.42	1	4731	C16 H16 N2 Na O2	(M+Na)+

--- End Of Report ---

HRMS spectrum of hBN8

hBN9

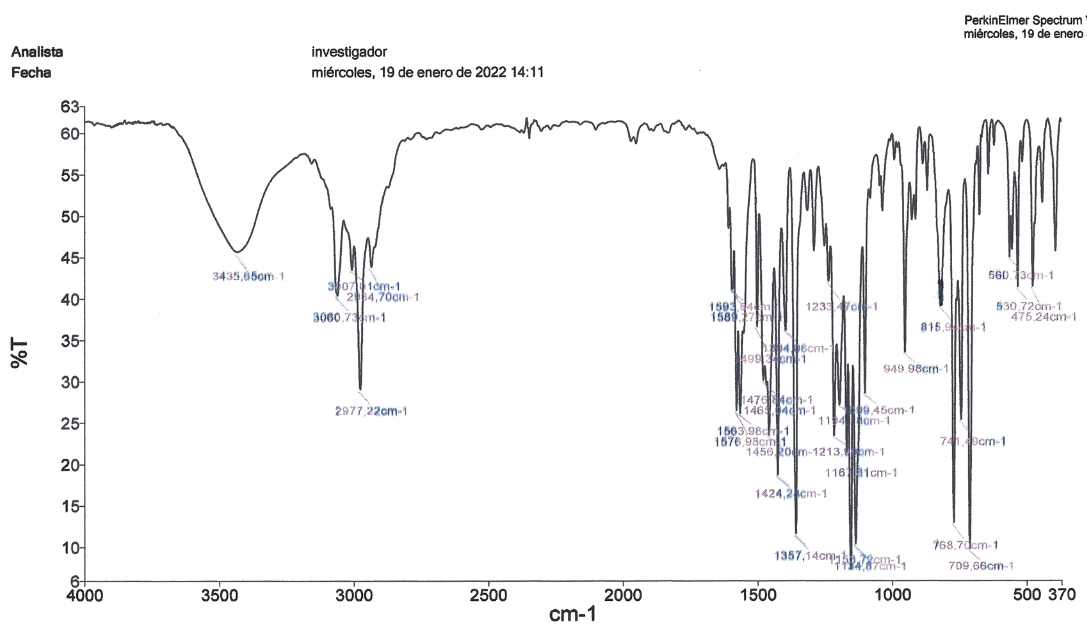

IR spectrum of hBN9

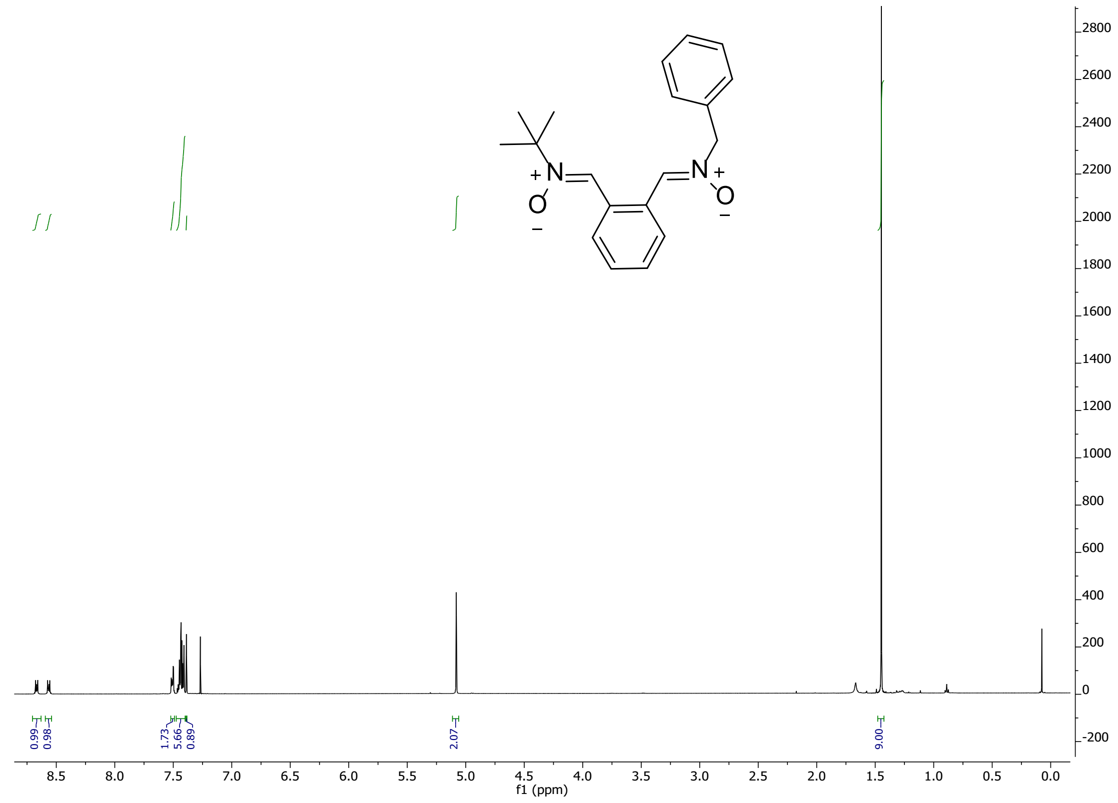

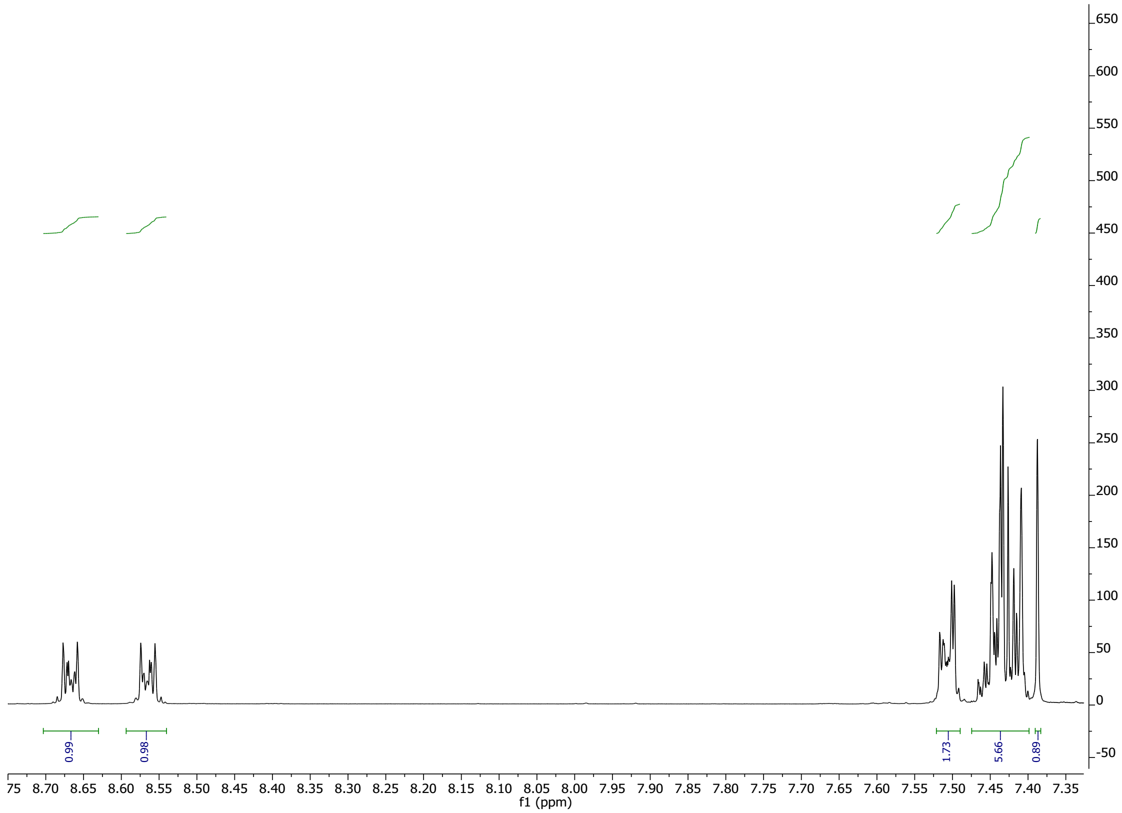

^{1}H NMR spectrum of hBN9

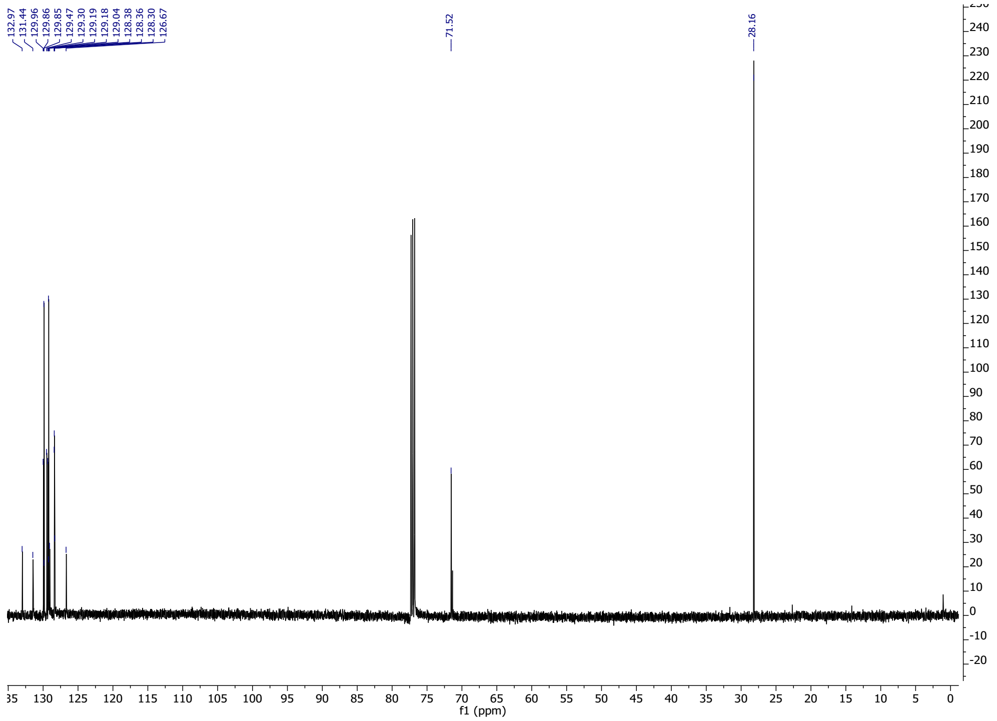

^{13}C NMR spectrum of hBN9

Qualitative Compound Report

**Data File**	14667_DDI_107_01.d	**Sample Name**	DDI_107
**Sample Type**	Sample	**Position**	Vial 6
**Instrument Name**	Instrument 1	**User Name**	
**Acq Method**	ESI_ACN_75_pos.m	**IRM Calibration Status**	Some Ions Missed
**DA Method**	Defecto_modificado_CS.m	**Comment**	

Compound Table

Compound Label	RT	Mass	Abund	Formula	Tgt Mass	Diff (ppm)
Cpd 1: C19 H22 N2 O2	0.87	310.16777	202792	C19 H22 N2 O2	310.16813	-1.14

Compound Label	RT	Algorithm	Mass
Cpd 1: C19 H22 N2 O2	0.87	Find By Formula	310.16777

MS Zoomed Spectrum

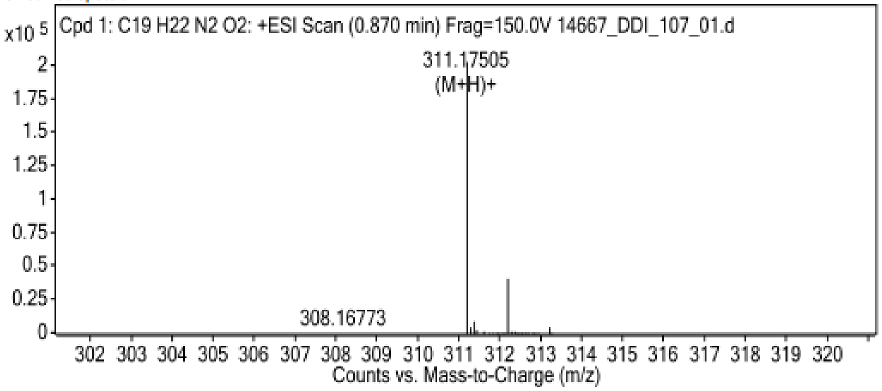

MS Spectrum Peak List

m/z	*Calc m/z*	Diff(ppm)	Abund	Formula	Ion
311.17505	311.1754	-1.12	202792	C19 H23 N2 O2	(M+H)+
311.27194			5996		
311.33665			10339		
311.41604			3191		
311.59461			1735		
312.17788	312.17858	-2.25	41673	C19 H23 N2 O2	(M+H)+
312.26478			2143		
312.34159			2165		
312.39223			1280		
313.18098	313.18143	-1.43	5886	C19 H23 N2 O2	(M+H)+

--- End Of Report ---

HRMS spectrum of hBN9
